# Field study on the health status of children aged 0–18 in container cities after the 2023 Türkiye earthquake - Hatay example

**DOI:** 10.1186/s12889-026-26945-w

**Published:** 2026-03-18

**Authors:** Ecem Gülistan Çakar, Emel Demir

**Affiliations:** Pediatric Nursing, Hatay Mustafa Kemal University, Hatay, Türkiye

**Keywords:** Türkiye, Hatay, Eartquake, Children, Container Cities, Children’s health; Post-disaster health

## Abstract

**Background:**

In Hatay, Türkiye, which suffered extensive devastation from the earthquake of 2023, people were living in container cities during the recovery process. While the physical and psychological effects of the earthquake continued, children faced health risks in their new living spaces. This study was conducted to assess the health status of children aged 0–18 living in Hatay temporary shelters following the earthquake.

**Methods:**

This comparative, cross-sectional, descriptive study was conducted between August and December 2023 with 1013 children living in container cities who met the study criteria. Age-appropriate health assessment forms, the State-Trait Anxiety Inventory, and the Post-Traumatic Stress Response Scale were used in the study. The obtained data were analyzed using SPSS. Descriptive statistics are expressed as mean and standard deviation, and categorical variables are expressed as frequency (n) and percentage (%). The Mann-Whitney U test, Kruskal-Wallis test and Post-Hoc tests were applied to the variables. Relationships between categorical variables were analyzed using the Chi-square test and, when necessary, the Fisher Exact test.

**Results:**

The study found that children’s health status in the post-disaster period varied significantly based on variables such as age group, gender, time spent in the container city, and the extent of damage to their homes. In physical health, nutritional problems and infectious diseases (1. Upper respiratory tract infection, 2. acute gastroenteritis, 3. skin diseases) were the most prevalent across age groups. Half of children aged 0–2 (50%), 51.7% of children aged 3–11, and 61.3% of adolescents aged 12–18 lost at least one family member in the earthquake. In mental health, stress and behavioral changes, state and trait anxiety, and PTSD were identified, particularly in adolescents.

**Conclusions:**

The results revealed that children living in post-disaster container cities were affected multidimensionally in terms of physical development, nutrition, vaccination, hygiene, and mental health. In the post-disaster recovery phase, planning and developing child health-centered services are essential.

**Supplementary Information:**

The online version contains supplementary material available at 10.1186/s12889-026-26945-w.

## Background

Millions of children in different parts of the world are exposed to natural disasters every year, and children are among the most affected and vulnerable groups [1]. Due to their ongoing growth and development processes, limited coping skills, and dependence on caregivers, children are more susceptible to the physical, psychological, and social effects of disasters compared to adults. Furthermore, the anatomical, physiological, immunological, and developmental differences between children and adults make them a more fragile group in the face of disasters [1–3]. Following the earthquakes centered in Kahramanmaraş on February 6, 2023, it was reported that 21.3% (4,805,662) of children aged 0–17 were directly affected by the disaster, experiencing serious disruptions in access to safe shelter, nutrition, healthcare services, and education [4]. Due to severe damage or destruction of homes following the earthquakes and ongoing aftershocks, many families were left homeless; children and their families were first placed in tent areas and then in container cities established as a temporary housing solution [5]. The United Nations Office for the Coordination of Humanitarian Affairs (OCHA) reported in its 2023 Earthquake Situation Report that approximately 2.5 million children are in need of humanitarian assistance and approximately 2.4 million people are living in temporary settlements [6]. While container cities function as temporary living spaces providing shelter and security, they also pose multifaceted health and developmental risks for children due to crowded living conditions and environmental limitations [7]. The sudden change in living arrangements and inadequate hygiene conditions have made it difficult for children to maintain proper nutrition, sleep, privacy, and healthy social relationships [8, 9].Studies have shown that in the post-earthquake period, children experience difficulties accessing clean water and hygiene products, nutritional problems and infectious diseases increase, and there are significant increases in sleep problems and anxiety levels [10, 11].

The deterioration of basic living conditions during disasters causes multidimensional physical, psychological, and social impacts on children, one of the most vulnerable segments of society. Accompanying inadequacies in shelter, nutrition, and hygiene, traumatic events, and the lack of psychosocial services seriously jeopardize children’s health [12]. The 2023 earthquake in Türkiye, one of the most devastating disasters that caused damage across 11 provinces, affected approximately 9.1 million people, including 4 million children. In the post-earthquake period, 1,915 unaccompanied children were identified, and 1,661 of them were reunited with their families. Furthermore, a UNICEF report dated March 2023 stated that basic necessities such as hygiene kits, winter clothing, heaters, and blankets were delivered to 351,381 people, including 200,277 children. On the other hand, in February 2023, UNICEF provided psychological first aid support to 5,100 children in five provinces (Gaziantep, Kilis, Mardin, Diyarbakır and Şanlıurfa), but it was reported that Hatay was not among these provinces [13]. Hatay’s exclusion from the list of provinces is related to the widespread destruction caused by the earthquake, the serious damage to transportation and infrastructure lines, the high number of affected populations, and the resulting difficulties in delivering humanitarian aid to those in need in a timely and sufficient manner [6].

According to Turkish Statistical Institute (TÜİK) data (2024), the prevalence of underweight in children aged 0–4 years in Türkiye is reported as 1.7%, the prevalence of stunting as 6.0%, the rate of underweight as 1.5%, and the rate of overweight as 8.1% [14]. According to OECD data, 20% of 15-year-old children in Turkey skip at least one meal a week, and one in five children goes to school hungry [15]. Skipping meals, especially neglecting breakfast, is among the most common negative eating habits in children and adolescents. In this context, to support adequate and balanced nutrition for children in Türkiye, it is recommended that meals not be skipped, that food diversity be increased, that whole grains, fruits and vegetables, and dairy products be consumed regularly, that sugary and processed foods and sugary drinks be limited, and that sufficient water consumption be encouraged [16]. In disasters and similar extraordinary situations, the decrease in food security, economic losses, difficulties in accessing clean drinking water and inadequate hygiene conditions are among the main factors that negatively affect the nutritional status of children [17]. In the early period after a disaster, these effects mostly manifest as acute nutritional problems (weight loss, weakness, skipping meals), while if the process is prolonged, permanent consequences such as chronic nutritional problems and growth retardation can be seen [18].

Feeding infants and young children who are dependent on adult care is a priority; breastfeeding is considered the most appropriate method of infant nutrition and is generally the safest option during disaster conditions [19]. However, in the post-disaster period, multidimensional factors such as lack of privacy, lack of appropriate breastfeeding spaces, inadequate social support, mother-infant separation, lack of information, high stress levels, and feelings of insecurity can negatively impact breastfeeding continuation [20, 21]. A field study conducted after the 2010 Haiti earthquake reported poor breastfeeding practices in shelters [22]. Such conditions may lead parents to use formula, but due to poor hygiene, difficulty accessing clean water, and the lack of proper storage conditions in disaster settings, formula use can increase the risk of infection and threaten infant health [19]. The nutrition of children in other age groups is often overlooked during disasters. Following the 2023 Türkiye Earthquake, new data has reported that inadequate nutritional content, access to clean drinking water, uncontrolled distribution of unhealthy foods, and the repetitive presentation of food types negatively impact child nutrition [23].

After a disaster, children become extremely vulnerable to infectious diseases due to their underdeveloped immune systems. The World Health Organization defines immunization as the process of protecting individuals from infectious diseases through vaccines and emphasizes that vaccination should not be interrupted during disaster conditions [24]. During this period, children’s crowded living spaces, inadequate hygiene habits, and difficulties accessing clean water and food sources lead to increased hospitalization rates, including for infectious diseases. Studies on the earthquakes in Lushan, Nepal, and Japan reported that respiratory infections, skin diseases, diarrhea, trauma, and chronic disease symptoms were common in children [25–28]. A study conducted in the emergency department after the earthquake in Türkiye reported that the most common illnesses seen in children were acute upper respiratory tract infection, acute gastroenteritis, and otitis media [10]. More than three-quarters of post-disaster mortality rates are due to infectious diseases [29]. Deaths from infectious diseases during disasters are often preventable with timely and effective health services [17]. Therefore, if children’s vaccination records are unavailable, they should be considered unvaccinated based on their age, a new program should be established, and any unfinished vaccinations should be resumed [30]. Routine vaccinations against 13 diseases are provided within the scope of the childhood immunization program in Türkiye, and vaccination against fecal-oral diseases, such as hepatitis A and polio, should not be interrupted during disasters [31]. Additionally, measles, tetanus, pneumococcal, chickenpox, H. influenza type b, and meningococcal vaccines are recommended in risk areas [32]. On the other hand, deterioration of hygiene conditions in the post-disaster period accelerates the spread of infectious diseases, and inadequate access to clean water and toilet hygiene has serious impacts on children’s health. Studies conducted after the 2011 Great East Japan Earthquake found that clean water was the most affected resource, while toilet hygiene negatively impacted the health of disaster victims [33]. Lack of hygiene affects not only children’s physical health but also their psychological well-being, increasing anxiety and stress levels in environments where privacy cannot be ensured [34]. Therefore, continuity of health services, uninterrupted immunization practices, and ensuring hygiene conditions should be considered key components in protecting children’s physical and psychological health in the post-disaster period.

After disasters, children who are exposed to losses and difficult living conditions experience mental health problems, and individuals in each age group may react differently to these situations [35]. In the 0–3 year age group, reactions such as surprise, appearing anxious, desire to not be separated from their parents, bedwetting, speech disturbances, sleep disorders, decreased relationships with the environment, shyness or uncontrolled irritability, playing games that remind them of the disaster, insecurity, constant crying, and tics can be observed [36]. In the 4–6 year age group, symptoms such as bedwetting, thumb sucking, excessive devotion to parents, anxiety, fear, sleep disorders, games that remind them of the disaster, nightmares, repeating words, tics, and constantly asking questions about the disaster can be observed [37]. In the 7–12 year age group, children may exhibit behaviors from previous developmental periods; reactions such as attention deficit, sleep disturbance, fear, distrust of parents, not going to school, decline in academic success and deterioration in peer relationships may occur [26]. Between the ages of 13 and 18, adolescents may experience symptoms such as lack of interest in activities, excessive irritability, negative thoughts about the future, attention deficit, speech and sleep problems, reluctance to go to school, tendency to be alone, tendency to smoke or use substances, post-traumatic stress disorder (PTSD), depression, and anxiety [36, 38]. Studies conducted after the 1999 Marmara earthquake also reported that children experienced post-traumatic stress disorder, depression, and sleep disorders [39, 40]. Studies following the 2008 earthquake in the Wechuan region of China found that PTSD symptoms were more severe in children rescued from rubble, injured, or who lost loved ones, and that symptoms such as sleep disturbances and the re-experiencing of traumatic memories persisted even three years later [41]. Another study conducted eight years after the same earthquake found that PTSD symptoms were higher in children living closer to the earthquake zone [42]. Similarly, a study conducted after the 2015 Nepal earthquake reported that PTSD symptoms were common in adolescents [43]. A study conducted after the 8.8 magnitude earthquake in Chile found that psychosocial impairments increased significantly in children exposed to severe earthquakes [44].

Ensuring the continuity of health services, strengthening immunization and nutrition programs, and establishing safe living spaces are essential in the post-disaster period. Post-disaster child health is affected by many interconnected factors, such as nutrition, infectious diseases, hygiene, and mental well-being. This demonstrates that child health in disaster management should be addressed not only through clinical care but also from a holistic public health perspective. Disaster management encompasses pre-disaster preparedness, risk reduction and damage prevention, disaster response, and post-disaster recovery and reconstruction [45]. Post-earthquake field surveys play a key role in determining the priority and quality of services by identifying deficiencies in the field during the recovery and reconstruction processes. This study aims to examine the physical and mental health status of children living in temporary shelters in Türkiye following the 2023 earthquake, revealing their current situation in terms of nutrition, vaccination status, illness and hospitalization, hygiene conditions, and psychosocial impacts.

## Methods

### Study design and setting

This study is designed as a comparative, cross-sectional, and descriptive study. The cross- sectional and descriptive design was chosen to assess the current health status of children living in temporary shelters after the 2023 earthquake. In the comparative dimension of the study, the health status of children was compared according to age groups (0–2, 3–11, and 12–18 years); differences in anthropometric measurements, nutritional status, hygiene conditions, vaccination status, disease status, and hospitalization history were examined.

The study was conducted during the recovery process that began following the completion of the emergency response phase after the earthquakes that occurred in Kahramanmaraş on February 6, 2023. Research data were collected between August and December 2023.

This period represents a critical time for assessing the nutritional status, hygiene conditions, immunization status, history of illness and hospitalization, and psychosocial conditions (anxiety levels, post-traumatic stress responses, and emotional/behavioral problems) of children living in container settlements.

The study was conducted in temporary shelters (container cities) equipped with a Family Health Center located in the Antakya district of Hatay province. Currently, the Hatay Provincial Health Directorate records list 70 container cities in Hatay. Container cities are typically 21–30 m² metal module structures with one or two rooms, a small kitchen, and a bathroom. Laundries are planned as shared spaces in these living spaces, and families provide their own meals. Each container city where data was collected has a Family Health Center, providing psychosocial support and treatment services. It also includes activity areas to support social integration for children and families.

### Study population and sample size calculation

The study population consisted of 6,552 children aged 0–18 living in temporary shelters (container cities) in the Antakya district of Hatay province between August and December 2023. There are 70 container cities in Hatay. The child population in the Antakya district of Hatay province is 6,552. A convenient sampling method was used in sample selection. The study sample consisted of 1,013 children, based on the inclusion criteria. Furthermore, the minimum sample size required was calculated using the sample calculation formula below, when the number of individuals in the population is known [46]. Based on this formula (for z = 1.96; Alpha = 0.5; H = 0.05), the minimum sample size required was determined to be 323.

The following formula was used to calculate the sample size;

$$\begin{array}{l}\mathrm{n}=\frac{\mathrm{N}.\mathrm{z}^{2}.\upsigma^{2}}{\left(\mathrm{N}-1\right).\mathrm{H}^{2}+\mathrm{z}^{2}.\mathrm{p}^{2}}\\\mathrm{n}=\text{N. t. t. p. q}\;/\;\text{d. d.}\;\left(\mathrm{N}-1\right)+\;\text{t. t. p. q}\\\mathrm{n}=6552.\;1,96.\;1,96.\;0,33.\;0,67/0,05.\;0,05.\;\left(6552-1\right)+1,96.\;1,96.\;0,33.\;0,67=323\end{array}$$

N: Number of individuals in the study.

t: 1.96 (Alpha = Theoretical value found from t table at 0.05 infinite degrees of freedom.)

*p* = 0.33 (probability of occurrence / event occurrence proportion).

q = 0.67 (probability of non-occurrence / event non-occurrence proportion).

d: 10 (The desired ± deviation according to the frequency of occurrence of the event).

Research inclusion criteria:

- Living in Hatay/Antakya Temporary Accommodation Centers between August and December 2023,
- Being a child between the ages of 0–18 and experiencing an earthquake,

Exclusion criteria from the study:

- Living daily (being a guest) in designated container cities between August and December 2023,
- Families with children between the ages of 0–18 who did not experience the earthquake but were staying in the container city were excluded from the study.

### Data collection tools

Data were collected using child health assessment questionnaires and two scales developed by the researcher based on literature and specific to three different age groups. he researcher marked the responses to the questions asked face-to-face in the container cities, and the interviews lasted an average of 20 min. Following this, the child’s anthropometric measurements (height and weight) were taken. Height was measured using a portable, demountable, standing height meter. Weight was measured using a digital baby scale for infants and a Tanita scale for children and adolescents.

The survey forms consist of sociodemographic questions containing information about the household and the child, as well as questions designed to determine health status (nutritional status, anthropometric measurements, vaccination status, mental health status, and hygiene status). The questions used to determine health status vary for each age group.

Because the sample covered a wide age range (0–18 years), the health assessment forms were structured in an age-specific manner, taking into account the developmental characteristics of the children. While common variables such as sociodemographic characteristics, vaccination status, history of illness and hospitalization, and anthropometric measurements were retained in the forms for each age group; questions regarding nutrition, hygiene, and mental health were differentiated to suit the relevant developmental stage. In this context, in the 0–2 age group, nutritional assessment was addressed through indicators such as breastfeeding status, duration of breastfeeding, timing of the introduction of complementary foods, and feeding frequency while in the 3–11 and 12–18 age groups, age-appropriate nutritional habits and food diversity of children and adolescents were investigated. Similarly, hygiene and mental health assessments were structured in a way that was appropriate to the cognitive and developmental level of each age group. This approach supported the validity and reliability of the analyses by ensuring that the unique characteristics of each developmental stage were taken into account.

#### 0–2 year-old child health assessment questionnaire form

The questionnaire for this age group consists of questions designed to determine the health status of children. The form’s sociodemographic characteristics, vaccination status, illness and hospitalization history, and anthropometric measurements sections are common to all age groups. However, the nutritional status, mental health status, and hygiene status questions are structured differently for this age group. Detailed questions regarding the duration of breastfeeding, the timing of introducing complementary foods, feeding frequency, and growth and development indicators were also added. Additionally, psychosocial status and hygiene habits were assessed through parental observation. The form consists of a total of 76 questions [22, 25, 27, 47].

#### 3-11-year-old child health assessment questionnaire form

This age group includes common questions, plus additional questions in the nutritional, hygiene, and mental health sections, tailored to their age. These questions examine school-age children’s eating habits and personal hygiene behaviors (handwashing, toothbrushing, and access to clean water). The mental health section includes age-specific observation questions such as anxiety, sleep patterns, and post-traumatic behavioral changes. The form consists of a total of 78 questions [26, 48–51].

#### 12–18 year-old child health assessment questionnaire form

While the nutritional status and hygiene questions for adolescents are the same as for the 3–11 age group, the mental health questions are differentiated for each age group. This section addresses changes in adolescents’ emotional states, anxiety levels, and sleep and social relationships. The form consists of a total of 79 questions [25, 41–44, 52].

#### Spielberger State-Trait Anxiety Inventory

The inventory is a 40-item Likert-type scale developed by Spielberger and colleagues in 1970 to measure momentary (state) and general (trait) anxiety levels of individuals and adapted to Turkish by Öner and Le Compte (1983) [53, 54]. Twenty items of the scale assess state anxiety and 20 items assess trait anxiety. There are 10 reverse-scoring items in the state subscale and 7 reverse-scoring items in the trait subscale. Higher scores indicate higher anxiety levels. Possible scores from the scales range from 20 to 80. Cronbach’s alpha coefficients for the Turkish form were found to be 0.94–0.96 for the state subscale and 0.83–0.87 for the trait subscale. In our study, Cronbach’s alpha coefficients were found to be 0.876 for the state subscale and 0.857 for the trait subscale. Although the scale was initially designed for individuals aged 14 and over [53], several studies in the literature have shown that it is also valid and reliable in children and adolescents aged 12 and over. In this context, studies by Salim and Certel (2025), Duman (2008), and Karabulut et al. (2013) reported that the Spielberger State-Trait Anxiety Inventory is applicable and provides reliable results in samples aged 12 and over [55–57]. Accordingly, this scale was used in this study to assess the anxiety levels of adolescents aged 12–18 years.

#### Post-traumatic stress response scale for children

The Child Posttraumatic Stress Reaction Scale (PTSD-TS) was developed by Pynoos and colleagues in 1987, and its Turkish validity and reliability study was conducted by Erden et al. and colleagues [58, 59]. It is a 20-item scale administered in a semi-structured interview format, designed to assess symptoms of posttraumatic stress disorder (PTSD) in children following various traumatic events such as natural disasters, wars, life-threatening illnesses, or abuse. Each item is rated on a scale of 0–4 according to symptom severity. A total score between 0 and 11 indicates no or suspected PTSD; a score between 12 and 24 indicates mild PTSD; 25–39 indicates moderate PTSD; 40–59 indicates severe PTSD; and 60–80 indicates very severe PTSD. The reliability coefficients obtained by the test-retest method of the scale were found to be 0.86, and Cronbach’s alpha was 0.75. Inter-rater consistency was Kappa 0.87. In our study, the Cronbach’s alpha coefficient for the Post-Traumatic Stress Response Scale for Children was found to be 0.855. However, there are studies in the literature that use the scale in child and adolescent samples up to 18 years of age [60, 61]. Accordingly, the scale was applied to the 12–18 age group in this study.

### Statistical analysis

IBM SPSS 25 program was used in data analysis, and *p* < 0.05 was accepted as the significance level. Mean and standard deviation are given in the descriptive statistics of continuous variables; frequency (n) and percentage (%) are given in the description of categorical variables. Normality assumptions of variables were examined using the Kolmogorov-Smirnov test. The Mann-Whitney U test was used for comparisons of continuous variables that did not show a normal distribution between two groups, and the Kruskal-Wallis test was used for comparisons between three or more groups. If a significant difference was obtained as a result of the Kruskal-Wallis test, post-hoc tests were performed to determine which groups the difference originated from. Relationships between categorical variables were analyzed using Chi-square/Fisher exact analysis.

## Results

### Sociodemographic findings

Research data were collected from three different container cities. According to the socio-demographic distribution of the 1013 children included in the study, 55.3% were girls, the mean age was 11.23 ± 4.74, and by age group, 7.5% were children aged 0–2, 35.3% were children aged 3–11, and 57.2% were children aged 12–18. Considering the order of the children in the family, 39.1% were the first child (min = 1, max = 6). The proportion of children who did not attend school after the earthquake was 13%. 39.9% of the children are currently attending field schools. 0.9% of the children lost their fathers and 0.2% lost their mothers in the earthquake. 48.9% of the children settled in the container cities within the first month, 31.5% within 2–4 months, and 19.6% after 5 months. 54.7% of families in the container reported living with more than four people. The damage distribution of homes indicated that 21.2% of their homes were destroyed, 64.8% were severely damaged, 7.8% had moderate damage, 5.4% had minor damage, and 0.8% were undamaged. The distribution of parents’ educational background revealed that 3% were illiterate, 3.6% were only literate, 41.0% were primary school graduates, 29.1% were secondary school graduates, 18.9% were high school graduates, and 4.4% were university graduates. 50.8% of parents lost their jobs after the earthquake, and 50.6% experienced a decrease in their income (see Supplementary Table 1).

### Physical health

#### Findings regarding children’s anthropometric measurements

Pre-earthquake data were collected for the 0–18 age group and post-earthquake height and weight measurements were made. The pre- and post-earthquake values ​​cover a 6-month period. The average weight of the participants in the 0–2 age group before the earthquake was found to be 8.11 ± 2.85, and the average weight after the earthquake was measured as 10.61 ± 2.50. The average height for this age group before the earthquake was found to be 70.42 ± 8.63, and the average weight after the earthquake was measured as 78.28 ± 6.52. The average weight for the participants in the 3–11 age group before the earthquake was found to be 24.86 ± 8.82, and the average weight after the earthquake was measured as 27.04 ± 13.14. The average height for this age group before the earthquake was found to be 118.66 ± 16.99, and the average weight after the earthquake was measured as 122.05 ± 15.04. The average pre-earthquake weight of participants in the 12–18 age group was found to be 52.16 ± 10.57, and the average post-earthquake weight was measured as 53.81 ± 10.76. The average pre-earthquake height of this age group was measured as 158.29 ± 10.51, and 159.31 ± 10.02 after the earthquake (see Table 1). The post-earthquake average weight of girls was found to be 39.97 ± 16.39 kg, and of boys was found to be 42.51 ± 21.67 kg. The post-earthquake average height of girls was found to be 139.36 ± 25.80 cm, and of boys was found to be 140.93 ± 29.38 cm (see Supplementary Table 2).

**Table 1:** Height and weight of children aged 0–18 years

	0–2 Age	Age Grupları 3–11 Age	12–18 Age
Pre- Earthquake Weight	8.11 ± 2.85	24.86 ± 8.82	52.16 ± 10.57
Post- Eathquake Weight	10.61 ± 2.50	27.04 ± 13.14	53.81 ± 10.76
Pre- Earthquake Height	70.42 ± 8.63	118.66 ± 16.99	158.29 ± 10.51
Post- Earthquake Height	78.28 ± 6.52	122.05 ± 15.04	159.31 ± 10.02

#### Findings on children’s nutritional status

Nutrition information for children aged 0–18 has been compiled according to age. Data on breastfeeding for children aged 0–2 are presented. Before the earthquake, 75% of children were breastfeeding. After the earthquake, 89.5% of breastfed children continued breastfeeding, but 10.5% weaned, and 40.4% of children experienced a decrease in breastfeeding frequency. Among the reasons for the decrease in breastfeeding frequency, 65.3% reported decreased milk supply, 13% due to the mother’s illness, 17.4% due to the baby’s reluctance, and 4.3% due to the baby’s illness. The pre-earthquake prevalence of formula feeding among children aged 0–2 was 28.9%. After the earthquake, 18.2% of those using formula reported difficulties accessing formula. The percentage of those experiencing problems accessing clean water for formula preparation was 9.1%, and 36.4% were unable to provide hygienic conditions. The rate of inability to obtain age-appropriate complementary foods in the container city was 92.1%. In cases where their child wasn’t eating supplementary food, 45.4% of parents gave up, while 27.3% took a break and tried again. There were no children in container cities receiving support from a nutritionist, and the proportion of children receiving nutritional support after the earthquake was 26.3% (see Table 2).

**Table 2:** Nutritional status of children aged 0–2 years

	Sample size *n*	Percentage %
Pre- Earthquake Breatfeeding Status		
Yes	57	75.0
No	19	25.0
Continuation of Breastfeeding		
Yes	51	89.5
No	6	10.5
Change in Feeding Frequency After the Earthquake		
Yes	23	40.4
No	34	59.6
Reasons for Decreased Feeding Frequency		
Decreased Milk Supply	15	65.3
Infant Refusal	4	17.4
Infant Illness	1	4.3
Maternal Illness	3	13.0
Pre- Earthquake Formula Use		
Yes	22	28.9
No	54	71.1
Access to Formula Among Formula Users		
Yes	4	18.2
No	18	81.8
Access to Clean Water for Formula Preparation		
Yes	2	9.1
No	20	90.9
Ensuring Hygienic Conditions During Formula Preparation		
Yes	14	63.6
No	8	36.4
Reasons for Inability to Maintain Hygiene		
Difficulty Accessing Drinking Water	6	75.0
Lack of Confidence in Water Safety	2	25.0
Food Allergy		
Yes	0	0.0
No	76	100.0
Provision of Age-Appropriate Complementary Foods		
Yes	6	7.9
No	70	92.1
Approach Used When the Infant Refuses Complementary Food		
Stopped	5	45.4
Paused and Tried Again	3	27.3
Used Play-Based Feeding	3	27.3
Provision of Nutritional Support After the Earthquake		
Yes	20	26.3
No	56	73.7
Total	76	100.0

Balanced nutrition status: The rate of not being able to have a balanced diet was found to be 33.2% in children aged 3–11, and 16.8% higher in the 12–18 age group (*p* < 0.001). When the rate of decreased appetite was examined, it was found to be 20.1% in the 3–11 age group, and 8.8% higher compared to the 12–18 age group (*p* < 0.001). When the status of skipping meals was examined (due to reasons such as eating junk food, loss of appetite, neglect, difficulties in reaching meals), the rate of skipping meals was found to be 19.6% in the 3–11 age group, and 8.6% higher compared to the 12–18 age group (*p* < 0.001). The consumption of milk and dairy products was found to be 14% in the 3–11 age group, and 8.3% higher compared to the 12–18 age group (*p* = 0.006). Consumption of meat, fish, and chicken was found to be 14.8% higher in the 3–11 age group and 9.3% higher than in the 12–18 age group (*p* < 0.05). When the decrease in fruit and vegetable consumption was examined, this rate was found to be 7.3% higher in the 3–11 age group and 3.6% higher than in the 12–18 age group (*p* < 0.05). When bread consumption was examined, the rate of participants who answered yes was 97.8% in the 3–11 age group and 99.5% higher in the 12–18 age group (*p* < 0.05) (see Table 3). 

**Table 3:** Nutritional status of children aged 3–11 and 12–18 years

	3–11 Age		Age Groups 12–18 Age		
	Sample size *n*(358)	Percentage %	Sample size *n*(579)	Percentage %	*p*
Adequate and Balanced Nutrition					< 0.001*
Yes	239	66.8	482	83.2	
No	119	33.2	97	16.8	
Post-Earthquake Decreased Appetite					< 0.001*
Yes	72	20.1	51	8.8	
No	286	79.9	528	91.2	
Skipping Meals					< 0.001*
Yes	70	19.6	50	8.6	
No	288	80.4	529	91.4	
Excessive Junk Food Consumption^a^					0.974*
Yes	25	35.7	18	36.0	
No	45	64.3	32	64.0	
Loss of Appetite^a^					0.332*
Yes	48	68.6	30	60.0	
No	22	31.4	20	40.0	
Meal Omission^a^					0.417**
Yes	0	0.0	1	2.0	
No	70	100.0	49	98.0	
Lack of Age-Appropriate Meals^a^					0.075**
Yes	5	7.1	0	0.0	
No	65	92.9	50	100.0	
Lack of School Meals^a^					0.570**
Yes	1	1.4	2	4.0	
No	69	98.6	48	96.0	
Increased Junk Food Consumption					0.924*
Yes	249	69.6	401	69.3	
No	109	30.4	178	30.7	
Preparation of Special Meals					0.554*
Yes	341	95.3	545	94.1	
No	17	4.7	34	5.9	
Decrease in Milk and Dairy Product Consumption					0.006*
Yes	50	14.0	48	8.3	
No	308	86.0	531	91.7	
Decrease in Meat, Fish, and Poultry Consumption					0.010*
Yes	53	14.8	54	9.3	
No	305	85.2	525	90.7	
Decrease in Fruit and Vegetable Consumption					0.013*
Yes	26	7.3	21	3.6	
No	332	92.7	558	96.4	
Decrease in Bread Consumption					0.026*
Yes	8	2.2	3	0.5	
No	350	97.8	576	99.5	

^ a^Percentages were calculated based on participants who reported skipping meals (n=70 for ages 3–11; n=50 for ages 12–18)

*Chi-square analysis

**Fisher’s Exact test; *p*: Significance level; *p* < 0.05 was considered statistically significant

#### Findings regarding children’s vaccination status

When the status of children having vaccination cards was examined, it was seen that 1.4% of the 12–18 age group did not have vaccination card information, 35.5% in the 0–2 age group, and 81.3% in the 3–11 age group (*p* < 0.001) (see Supplementary 3). When the reasons for not having vaccination cards were examined, the status of missing in the earthquake was 91.1% in the 12–18 age group, 97.4% in the 0–2 age group, and 99.3% in the 3–11 age group (*p* < 0.001). The prevalence of vaccinations coming due after the earthquake was 0.9% in the 12–18 age group, 82.9% in the 0–2 age group, and 5.3% in the 3–11 age group (*p* < 0.001) (see Table 4).

**Table 4:** Vaccination status of children aged 0–18 years

	0–2 Age		3–11 Age		12–18 Age		*p*
	Sample size *n*(76)	Percentage %	Sample size *n*(358)	Percentage %	Sample size *n*(579)	Percentage %	
Presence of Vaccination Card							< 0.001*
Yes	49	64.5	67	18.7	8	1.4	
No	27	35.5	291	81.3	571	98.6	
Reason for Lack of Vaccination Card							< 0.001*
It Never Existed	1	3.7	2	0.7	51	8.9	
Lost During the Earthquake	26	96.3	287	99.3	522	91.1	
Completeness of Pre-Earthquake Vaccinations							0.118*
Fully Vaccinates	74	97.4	357	99.7	573	99.0	
Partially Vaccinated	2	2.6	1	0.3	6	1.0	
Due for Vaccination After the Earthquake							< 0.001*
Yes	63	82.9	19	5.3	5	0.9	
No	13	17.1	339	94.7	574	99.1	
Access to Vaccination							- ^**a**^
Yes	7	10.9	1	5.3	1	20.0	
No	57	89.1	18	94.7	4	80.0	

*Chi-square analysis

^a^*P*-value not reported because the assumptions of the Chi-square analysis were not met; *p*: Significance level; *p* < 0.05 was considered statistically significant

#### Findings regarding children’s hygiene status

According to hygiene status, 60.5% of parents of children aged 0–2 reported being able to maintain hygiene, while 39.5% reported being unable to do so. The most common reasons cited by families lacking hygiene were 30% inability to provide clean room air, 13.3% frequent water outages, 10% electricity outages, and 13.3% lack of hygiene supplies. 2.6% of parents reported inadequate individual hygiene care. Regarding the frequency of bathing their children, 23.7% reported bathing every day, 51.3% every other day, and 25% every three days. Regarding their children’s ability to provide age-appropriate shampoo, 3.9% reported being unable to provide age-appropriate shampoo, and 15.8% reported having difficulty providing their children with clean clothes (see Supplementary Table 4).

The hygiene status of participants aged 3–11 was compared with that of 12–18. The rate of not being able to maintain hygiene was found to be 60.3% in the 3–11 age group and 66.8% in the 12–18 age group (*p* < 0.05). The reason for not being able to maintain hygiene was stated as “Overcrowded family environment” by 79.6% in the 3–11 age group and 91.7% in the 12–18 age group (*p* < 0.05). The rate of “We have problems accessing water” as the reason for not being able to maintain hygiene was found to be 9.9% higher in the 3–11 age group and 1.6% higher in the 12–18 age group (*p* < 0.05). The rate of “I don’t have age-appropriate hygiene materials” as the reason for not being able to maintain hygiene was found to be 17.6% higher in the 3–11 age group and 3.11% higher in the 12–18 age group (*p* < 0.001). During this period, lice infestation was found to be 30.2% in the 3–11 age group and 16.6% in the 12–18 age group (*p* < 0.001). The rate of those who could not provide clean clothes was found to be 13.4% in the 3–11 age group and 2.8% in the 12–18 age group (*p* < 0.001). The rate of not being able to clean nails regularly was found to be 2.2% in the 3–11 age group and 0.5% in the 12–18 age group (*p* < 0.05). The rate of not being able to brush teeth regularly was found to be 44.4% in the 3–11 age group and 5.9% in the 12–18 age group (*p* < 0.05). The reasons for not being able to provide hygiene during this period, such as frequent water cuts, frequent electricity cuts, inability to provide clean room air due to building demolitions, and inability to access lice shampoo during this period, are not statistically significant according to the age distribution of 3–11 and 12–18 (*p* > 0.05) (see Table 5).

**Table 5:** Hygiene status of children aged 3–11 and 12–18 years

	3-11 Age		12-18 Age		*p*
	Sample sizen 358)	Percentage %	Sample sizen (579)	Percentage %	
Maintaining Hygiene					0.043*
Yes	216	60.3	387	66.8	
No	142	39.7	192	33.2	
Crowded Living Conditions					0.001**
Yes	113	79.6	176	91.7	
No	29	20.4	16	8.3	
Difficulty Accessing Water					0.001 **
Yes	14	9.9	3	1.6	
No	128	90.1	189	98.4	
Frequent Water Interruptions					0.697**
Yes	8	5.6	9	4.7	
No	134	94.4	183	95.3	
Frequent Power Interruptions					0.727**
Yes	4	2.8	4	2.1	
No	138	97.2	188	97.9	
Insufficient Hygiene Supplies					<0.001*
Yes	25	17.6	6	3.1	
No	117	82.4	186	96.9	
Inability to Maintain Clean Indoor Air Due to Structural Damage					0.803*
Yes	36	25.4	51	26.6	
No	106	74.6	141	73.4	
Child’s Bathing Frequency					-^a^
Every day	106	29.6	112	19.3	
Every 2 Days	196	54.7	446	77.0	
Every 3 Days	52	14.5	21	3.6	
≥ 3 days	4	1.1	0	0.0	
Head Lice Infestation					<0.001*
Yes	108	30.2	96	16.6	
No	250	69.8	483	83.4	
Access to Anti-Lice Shampoo					0.377*
Yes	16	14.8	10	10.6	
No	92	85.2	84	89.4	
Ability to Maintain Clothing Cleanliness					<0.001*
Yes	310	86.6	563	97.2	
No	48	13.4	16	2.8	
Ability to Maintain Nail Cleanliness					0.026**
Yes	350	97.8	576	99.5	
No	8	2.2	3	0.5	
Regular Toothbrushing					<0.001*
Yes	199	55.6	545	94.1	
No	159	44.4	34	5 .9	

^a^ *P*-value not reported because the assumptions of the Chi-square analysis were not met; *p*: Significance level; *p* < 0.05 was considered statistically significant

*Chi-square analysis

**Fisher’s Exact test

#### Findings regarding children’s illness and hospitalization status

**Fig. 1:**
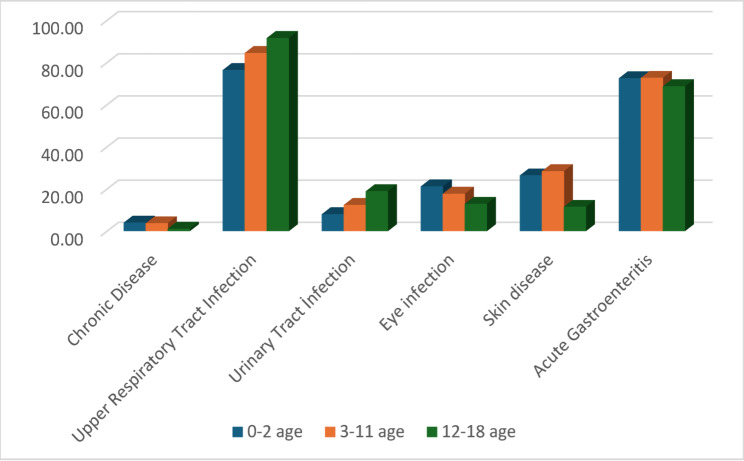
Diseases observed in children aged 0–18 years

Chronic disease status in children is 1% in the 12–18 age group, 3.9% in the 0–2 age group, and 3.6% in the 3–11 age group. In the post-earthquake period, the prevalence of acute upper respiratory tract infections was highest in the 12–18 age group, 84.4% in the 3–11 age group, and 76.3% in the 0–2 age group (*p* < 0.001). Acute gastroenteritis was high in the 0–2 age group, 72.4% in the 3–11 age group, 72.6% in the 12–18 age group, and 68.6% in the 12–18 age group; the difference between age groups was not statistically significant (*p* > 0.05). The prevalence of skin diseases was highest in the 3–11 age group, 28.8% in the 0–2 age group, 26.3% in the 0–2 age group, and 11.4% in the 12–18 age group (*p* < 0.001). Eye infection rates were highest in the 0–2 age group (21.1%), 17.6% in the 3–11 age group, and 12.8% in the 12–18 age group (*p* = 0.043). Urinary tract infection rates were highest in the 12–18 age group (18.8%), 7.9% in the 0–2 age group, and 12.3% in the 3–11 age group (*p* = 0.004). Household accidents, insect bites, and allergic reactions were rare (see Fig. 1) (see Table 6). 

**Table 6:** Illness and hospitalization status of children aged 0–18 Years

	0–2 Age		3–11 Age			12–18 Age	*p*
	Sample size *n*(76)	Percentage %	Sample size *n*(358)	Percentage %	Sample size *n*(579)	Percentage %	
Presence of Chronic Illness/ Special Needs							- ^**a**^
Yes	3	3.9	13	3.6	6	1.0	
No	7 3	96.1	345	96.4	573	99.0	
Difficulty Accessing Healthcare Services							- ^**a**^
Yes	1	1.3	15	4.2	12	2.1	
No	75	98.7	343	95.8	567	97.9	
Hospitalization Status							- ^**a**^
Yes	6	7.9	10	2.8	4	0.7	
No	70	92.1	348	97.2	575	99.3	
Acute Upper Respiratory Tract Infection							< 0.001*
Yes	58	76.3	302	84.4	530	91.5	
No	18	23.7	56	15.6	49	8.5	
Acute Gastroenteritis							0.384*
Yes	55	72.4	260	72.6	397	68.6	
No	21	27.6	98	27.4	182	31.4	
Urinary Tract Infection (UTI)							0.004*
Yes	6	7.9	44	12.3	109	18.8	
No	70	92.1	314	87.7	470	81.2	
Eye Infection							0.043*
Yes	16	21.1	63	17.6	74	12.8	
No	60	78.9	295	82.4	505	87.2	
Skin Disease							< 0.001*
Yes	20	26.3	103	28.8	66	11.4	
No	56	73.7	255	71.2	513	88.6	
İnjury							- ^**a**^
Yes	0	0.0	5	1.4	3	0.5	
No	76	100.0	353	98.6	576	99.5	
Animal Bite/ Sting							- ^**a**^
Yes	0	0.0	0	0.0	2	0.3	
No	76	100.0	358	100.0	577	99.7	
Domestic Accident							- ^**a**^
Yes	1	1.3	3	0.8	6	1.0	
No	75	98.7	355	99.2	573	99.0	
Reason							- ^**a**^
Fall	0	0.0	2	50.0	3	60.0	
Burn	0	0.0	0	0.0	2	40.0	
Poisoning	0	0.0	2	50.0	0	0.0	
Ingestion of Corrosive or Caustic Substances							
	1	100.0	0	0.0	0	0.0	
Allergic Disease							- ^**a**^
Yes	0	0.0	10	2.8	21	3.6	
No	76	100.0	348	97.2	558	96.4	
Increase in Symptoms							0.441**
Yes	-	-	6	60.0	8	38.1	
No	-	-	4	40.0	13	61.9	

^a^ *P* value not reported because the assumptions of the chi-square analysis were not met; *p*: significance level; *p*<0.05 was considered statistically significant

*Chi-square analysis; *Fisher’s Exact test

### Mental health

Half of the children aged 0–2 lost at least one family member in the earthquake. 2.6% of the children who lost a sibling lost a sibling, and 47.4% lost other family members. When the children’s emotional reactions after the earthquake were evaluated, anxiety was always observed in 9.2% and partially observed in 80.3%. When the children’s bond with their parents was evaluated, the desire to be together constantly was always observed in 59.2% and partially observed in 39.5%. Similarly, when separated from their parents, 48.7% of children always cried, and 50.0% cried partially. When the findings regarding sleep patterns were examined, it was determined that 51.3% of children experienced changes in their sleep patterns in the post-earthquake period. Only 3.9% of parents received any psychological support, while 96.1% did not (see Supplementary Table 5). No statistically significant relationship was found in the responses to mental health questions according to gender at ages 0–2 (*p* > 0.05) (see Supplementary Table 6).

The mean age for children aged 3–11 was 7.67 ± 2.42 (min = 3, max = 11). In this group, 51.7% of children lost at least one family member in the earthquake. Of the children who experienced loss, 0.3% lost their father, 1.4% lost their sibling, and 50.0% lost other family members. In the post-earthquake period, children’s anxious reactions were reported as always (16.2%), and partially (77.1%). Children’s desire to be with their parents was reported as always (38.3%), and partially (55.9%). Children’s feelings of distrust of their parents’ words were reported as always (3.9%), and partially (35.2%). Changes in children’s sleep patterns were observed in 53.4% ​​of children, while no change was observed in 46.6%. Additionally, the frequency of children waking up with nightmares was found to be 9.8% always and 54.2% partially. Children asking questions about earthquakes was found to be 23.5% always and 58.7% partially. Avoidance behaviors in children were found to be 1.7% always and 59.8% partially. The subcategories of avoidance behaviors were 55% always sleeping with their mothers, 8.9% having difficulty making friends or spending time with peers, 2% refusing to go to the hospital, 19.3% refusing to go to school, and 4.7% not participating in activities. Regression behaviors were reported in 27.1% of the children. Among the regression behaviors demonstrated were 6.1% thumb sucking, 55.7% bedwetting, 1.4% crawling, 5% baby talk, 0.8% refusing to be fed by someone else, 2% wanting to be carried constantly, 0.8% mispronouncing words they said correctly, and 4.7% wanting to suck a pacifier or breast. It was determined that 5.6% of parents received psychological support, while 94.4% did not receive any support (see Table 7 ). In the mental state assessment of children according to gender, regarding the state of distrust of what their parents say, the proportion of girls who answered “always” was 5.8% and 28.9%, respectively, while the proportion of boys who answered “always” was 1.8% and 42.3%, respectively (*p* < 0.05). Regarding questions about earthquakes, the proportion of girls who answered “always” was 24.7% and 62.1%, respectively, while the proportion of boys who answered “always” was 22% and 54.8%, respectively (*p* < 0.05). There were no statistically significant differences by gender in the other mental health questions (*p* > 0.05). (see Supplementary Table 7).

**Table 7:** Mental health status of children aged 3-11 Years by gender

	Female		Male		*p*
	Sample size n (190)	Percentage %	Sample size n (168)	Percentage %	
Loss of Family Members					0.969*
Yes	98	51.6	87	51.8	
No	92	48.4	81	48.2	
Family Member Lost					-^a^
Father	1	1.0	0	0.0	
Sibling	3	3.1	2	2.3	
Other Family Members	94	95.9	85	97.7	
Appearing Confused/Anxious					0.079*
Always	23	12.1	35	20.8	
Sometimes	153	80.5	123	73.2	
Never	14	7.4	10	6.0	
Wanting to Be Continuously with the Parent					0.150*
Always	80	42.1	57	33.9	
Sometimes	102	53.7	98	58.3	
Never	8	4.2	13	7.7	
Feeling Distrust Toward What the Parent Says					0.009*
Always	11	5.8	3	1.8	
Sometimes	55	28.9	71	42.3	
Never	124	65.3	94	56.0	
Change in Sleep Pattern					0.254*
Yes	96	50.5	95	56.5	
No	94	49.5	73	43.5	
Waking Up Due to Nightmares					0.474*
Always	22	11.6	13	7.7	
Sometimes	101	53.2	93	55.4	
Never	67	35.3	62	36.9	
Asking Questions About the Earthquake					0.046*
Always	47	24.7	37	22.0	
Sometimes	118	62.1	92	54.8	
Never	25	13.2	39	23.2	
Exhibiting Avoidance Behaviors					-**
Always	3	1.6	3	1.8	
Sometimes	118	62.1	96	57.1	
Never	69	36.3	69	41.1	
Consistently Co-Sleeping with the Mother					0.465**
Yes	110	90.9	87	87.9	
No	11	9.1	12	12.1	
Difficulty Making Friends					0.356**
Yes	20	16.5	12	12.1	
No	101	83.5	87	87.9	
Refusing to Go to the Hospital When Necessary					1.00**
Yes	4	3.3	3	3.0	
No	117	96.7	96	97.0	
Not Wanting to Go to School					0.249*
Yes	34	28.1	35	35.4	
No	87	71.9	64	64.6	
Not Participating in Activities					0.179**
Yes	12	9.9	5	5.1	
No	109	90.1	94	94.9	
Exhibiting Regression Symptoms					0.717*
Yes	53	27.9	44	26.2	
No	137	72.1	124	73.8	
Thumb Sucking					0.992*
Yes	12	22.6	10	22.7	
No	41	77.4	34	77.3	
Enuresis					0.836**
Yes	29	54.7	25	56.8	
No	24	45.3	19	43.2	
Crawling					0.805**
Yes	3	5.7	2	4.5	
No	50	94.3	42	95.5	
Baby Talk					0.097**
Yes	13	24.5	5	11.4	
No	40	75.5	39	88.6	
Not Eating Independently					
Yes	2	3.8	1	2.3	1.00**
No	51	96.2	43	97.7	
Wanting to Be Carried Constantly					1.00**
Yes	4	7.5	3	6.8	
No	49	92.5	41	93.2	
Speech Regression					0.249**
Yes	3	5.7	0	0.0	
No	50	94.3	44	100.0	
Wanting a Pacifier					0.877*
Yes	9	17.0	8	18.2	
No	44	83.0	36	81.8	
Receiving Psychological Support (Parent)					0.821**
Yes	10	5.3	10	6.0	
No	180	94.7	158	94.0	

^a^*P*-value not reported because the assumptions of the Chi-square analysis were not met; *p*: Significance level; *p* < 0.05 was considered statistically significant

*Chi-square analysis; **Fisher’s Exact test

The average age between 12 and 18 years is 14.73 ± 1.75 (min = 12, max = 18). 61.3% of adolescents in this age group lost at least one family member in the earthquake. Of the children who experienced loss, 0.3% lost their father, 0.2% lost their sibling, and 60.8% lost another family member. When the rate of adolescents experiencing anxiety in the post-earthquake period was examined, 5.4% reported “always” and 88.8% reported “partially.” The rate of adolescents not wanting to be alone was 7.1% reported “always” and 62.3% reported “partially.” The rate of adolescents lacking interest in normal activities was 7.9% reported “always” and 64.6% reported “partially.” Negative thoughts about the future were 21.2% reported “always” and 67.0% reported “partially.” It was found that 32.6% of adolescents had changes in their sleep patterns, while 67.4% had no changes. Nightmares were reported as always occurring in 6.9% of adolescents and partially occurring in 57.5%. Substance or cigarette use among adolescents was found to be 2.6%. When findings related to academic life were examined, 56.1% of adolescents showed a lack of interest in school, 43.2% had a decline in academic performance, and 11.6% exhibited aggressive behavior. It was determined that 9.3% of adolescents received some form of psychological support, while 90.7% did not (see Supplementary Table 8). In the mental state assessment of adolescents by gender, the proportion of participants who responded “always” to “appear anxious” was found to be 5.2% in girls and 92.1% in boys, and 5.6% in boys and 84.5% in boys. The rate of not feeling anxious was found to be 2.7% in girls and 10.0% in boys, the difference is statistically significant (*p* < 0.05). The rate of lack of interest in normal activities was found to be 8.5% in girls and 68.6% partly in girls and 7.2% always and 58.4% partly in boys. The rate of lack of interest in normal activities was found to be 22.9% in girls and 33.5% in boys, the difference is statistically significant (*p* < 0.05). The rate of waking up with nightmares was found to be 8.5% in girls and 64% partly in girls and 4.8% in boys and 49% in boys. The difference between the participants who answered “always” in the case of nightmares was statistically significant (*p* < 0.001). The rate of substance or cigarette use was found to be 0.9% in girls and 4.8% in boys (*p* < 0.05). There is no statistically significant difference in other mental health status questions according to gender (*p* > 0.05) (see Table 8).

**Table 8:** Mental Health Status of Adolescents Aged 12–18 Years by gender

	Female		Male		*p*
	Sample size *n*(328)	Percentage %	Sample size *n*(251)	Percentage %	
Loss of Family Members					0.399*
Yes	206	62.8	149	59.4	
No	122	37.2	102	40.6	
Family Member Lost					-^**a**^
Father	1	0.5	1	0.7	
Sibling	1	0.5	0	0.0	
Other Family Members	204	99.0	148	99.3	
Appearing Confused/Anxious					0.001*
Always	17	5.2	14	5.6	
Sometimes	302	92.1	212	84.5	
Never	9	2.7	25	10.0	
Wanting to Be Alone Constantly					0.232*
Always	25	7.6	16	6.4	
Sometimes	212	64.6	149	59.4	
Never	91	27.7	86	34.3	
Experiencing Lack of Interest in Normal Activities					0.018*
Always	28	8.5	18	7.2	
Sometimes	225	68.6	149	59.4	
Never	75	22.9	84	33.5	
Having Negative Thoughts About the Future					0.326*
Always	73	22.3	50	19.9	
Sometimes	222	67.7	166	66.1	
Never	33	10.1	35	13.9	
Change in Sleep Pattern					0.730*
Yes	109	33.2	80	31.9	
No	219	66.8	171	68.1	
Waking Up Due to Nightmares					< 0.001*
Always	28	8.5	12	4.8	
Sometimes	210	64.0	123	49.0	
Never	90	27.4	116	46.2	
Substance or Cigarette Use During This Period					0.004**
Yes	3	0.9	12	4.8	
No	325	99.1	239	95.2	
Experiencing Lack of Interest in School					0.851*
Yes	183	55.8	142	56.6	
No	145	44.2	109	43.4	
Decline in School Performance					0.657*
Yes	139	42.4	111	44.2	
No	189	57.6	140	55.8	
Exhibiting Aggressive Behaviors					0.802*
Yes	37	11.3	30	12.0	
No	291	88.7	221	88.0	
Receiving Psychological Support (Child)					0.684*
Yes	32	9.8	22	8.8	
No	296	90.2	229	91.2	

^a^*P*-value not reported because the assumptions of the Chi-square analysis were not met; p: Significance level; p < 0.05 was considered statistically significant

*Chi-square analysis; **Fisher’s Exact test

#### Findings on the Spielberg State-trait anxiety inventory of 12–18 year-old children

The state subscale scores of the State-Trait Anxiety Inventory for children aged 12–18 are shown in Table 9. The state anxiety score for boys was 39.34 ± 5.94, and for girls it was 38.52 ± 5.45. State anxiety scores differed significantly according to the duration of arrival to the container city (*p* = 0.007). The state anxiety scores of children who arrived at the container city for 5 months or later were 45.60 ± 6.82, for those who arrived within 1 month it was 39.37 ± 5.71, and for those who arrived within 2–4 months it was 38.47 ± 5.49. It was determined that the state anxiety scores of the children did not differ significantly according to the education level of the parents, the degree of damage to the house they lived in, the decrease in income, or the number of members of the household (*p* > 0.05) (see Table 9).

**Table 9:** Comparison of State Anxiety Subscale Scores of the State–Trait Anxiety Inventory for Children According to Sociodemographic Variables

Parametreler	Mean ± SD	Median (Min – Max)	*p*
Gender			0.135*
Female	38.52 ± 5.45	38.00 (25.00–56.00)	
Male	39.34 ± 5.94	39.00 (25.00–56.00)	
Groups			-
0–2	**-**	**-**	
3–11	**-**	**-**	
12–18	38.88 ± 5.68	38.00 (25.00–56.00)	
Time of Arrival to the Container Settlement			0.007**
≤ 1 month	39.37 ± 5.71	39.00 (25.00–56.00)	
2–4 Months	38.47 ± 5.49	38.00 (25.00–51.00)	
≥ 5 months	45.60 ± 6.82	45.00 (29.00–65.00)	
Parental Education			0.305**
Illiterate	36.08 ± 3.70	34.50 (32.00–45.00)	
Literate	38.42 ± 6.05	38.00 (29.00–51.00)	
Primary School	39.18 ± 5.80	39.00 (25.00–56.00)	
Secondary Scholl	38.89 ± 5.43	38.00 (28.00–56.00)	
High School	38.77 ± 5.67	38.00 (29.00–56.00)	
Unıversity Degree	37.18 ± 6.61	36.00 (25.00–49.00)	
Job Loss			0.356*
Yes	39.05 ± 5.74	38.00 (27.00–56.00)	
No	38.70 ± 5.61	38.00 (25.00–56.00)	
Reduction in Income			0.656*
Yes	38.93 ± 5.72	38.00 (27.00–52.00)	
No	38.83 ± 5.64	38.00 (25.00–56.00)	
House Damage Status			0.150**
Destroyed	37.78 ± 5.10	37.00 (29.00–50.00)	
Severely Damaged	39.23 ± 5.84	39.00 (25.00–56.00)	
Moderately Damaged	38.60 ± 5.48	38.00 (30.00–51.00)	
Slightly Damaged	38.20 ± 5.45	37.00 (29.00–46.00)	
Damaged	37.00 ± 0.00	37.00 (37.00–37.00)	
Household Size			0.195*
≤ 4	38.43 ± 5.30	38.00 (25.00–51.00)	
> 4	39.26 ± 5.96	38.50 (27.00–56.00)	
Type of School Attended			0.811**
Temporary Field School	38.57 ± 5.36	38.00 (27.00–56.00)	
Regular School	39.13 ± 5.81	38.00 (25.00–56.00)	
Open Education	39.38 ± 5.90	38.50 (31.00–51.00)	

*Mann Whitney test; **Kruskal Wallis test

For children aged 12–18, the trait anxiety score of the State-Trait Anxiety Inventory was found to be 46.42 ± 6.62 in girls and 43.51 ± 6.84 in boys (*p* < 0.001). According to the duration of arrival to the container city, the trait anxiety scores of the participants who arrived 5 months or later were found to be 42.07 ± 7.08, 45.31 ± 6.78 within 1 month, and 45.60 ± 6.82 within 2–4 months. The difference between the groups was statistically significant (*p* < 0.05). The highest mean score (Trait Anxiety score) was 45.81 ± 6.88 in children living in heavily damaged houses, and the lowest mean score was 41.60 ± 4.81 in children living in less damaged houses; the difference between the groups was statistically significant (*p* < 0.05). It was determined that children’s state anxiety scores did not differ significantly according to the education level of the parents, decrease in income and number of household members (*p* > 0.05) (see Table 10).

**Table 10:** Comparison of trait anxiety subscale scores of the state–trait anxiety inventory for children across sociodemographic variables

Parametreler	Mean ± SD	Median (Min – Max)	*p*
Gender			< 0.001*
Female	46.42 ± 6.62	46.00 (28.00–63.00)	
Male	43.51 ± 6.84	43.00 (28.00–65.00)	
Group			-
12–18 age	45.16 ± 6.86	44.00 (28.00–65.00)	
Time of Arrival to the Container Settlement			0.008**
≤ 1 month	45.31 ± 6.78	45.00 (28.00–63.00)	
2–4 Months	45.60 ± 6.82	45.00 (29.00–65.00)	
≥ 5 months	42.07 ± 7.08	42.00 (30.00–63.00)	
Parental Education			0.144**
Illiterate	41.33 ± 3.89	43.00 (33.00–46.00)	
Literate	42.32 ± 6.28	44.00 (32.00–54.00)	
Primary School	45.55 ± 6.95	45.00 (28.00–63.00)	
Secondary Scholl	45.24 ± 7.13	44.00 (30.00–65.00)	
High School	45.00 ± 6.23	46.00 (30.00–63.00)	
Unıversity Degree	45.12 ± 7.37	44.00 (35.00–61.00)	
Job Loss			0.802*
Yes	45.03 ± 6.91	45.00 (28.00–63.00)	
No	45.30 ± 6.82	44.00 (30.00–65.00)	
Reduction in Income			0.463*
Yes	44.89 ± 6.88	44.00 (28.00–63.00)	
No	45.43 ± 6.84	45.00 (30.00–65.00)	
House Damage Status			0.010**
Destroyed	43.58 ± 6.86	43.00 (29.00–63.00)	
Severely Damaged	45.81 ± 6.88	45.00 (28.00–65.00)	
Moderately Damaged	44.20 ± 6.39	44.00 (30.00–55.00)	
Slightly Damaged	41.60 ± 4.81	42.00 (31.00–49.00)	
Damaged	47.00 ± 9.90	47.00 (40.00–54.00)	
Household Size			0.913*
≤ 4	45.16 ± 6.70	45.00 (28.00–65.00)	
> 4	45.16 ± 7.01	44.00 (28.00–63.00)	
Type of School Attended			0.144**
Temporary Field School	44.36 ± 6.78	43.00 (28.00–63.00)	
Regular School	45.60 ± 6.85	45.00 (28.00–65.00)	
Open Education	43.13 ± 6.27	44.00 (32.00–50.00)	

*Mann Whitney test, **Kruskal Wallis test

#### Findings regarding post-traumatic stress response scale scores of 12–18 years old children

A comparison of the Post-Traumatic Stress Response Scale (PTSD) in children aged 12–18 according to some parameters is shown. When the children’s PTSD levels were examined by gender, 72% of girls and 28% of boys showed very severe PTSD. 66.2% of girls and 33.8% of boys showed severe PTSD, 47.9% of girls and 52.1% of boys showed moderate PTSD, and 43.5% of girls and 56.5% of boys showed mild PTSD. The distribution of children’s arrival time to the container city and PTSD levels was as follows: 52.0% of children with very severe PTSD arrived within 1 month, 44.0% within 2–4 months, and 4.0% within 5 months or later. In the severe PTSD group, 58.8% of children settled in the container city within 1 month, 36.8% within 2–4 months, and 4.4% within 5 months or later. In children with moderate PTSD symptoms, 58.8% came within 1 month, 28.4% within 2–4 months, and 12.9% after 5 months. In children with mild PTSD symptoms, this rate was 62.9% within 1 month, 30.6% within 2–4 months, and 6.5% after 5 months. According to the educational background of the parents, 44.0% of the parents of children with very severe PTSD symptoms were primary school graduates, 38.0% were secondary school graduates, 10.0% were high school graduates, and 6.0% were university graduates. Of the parents of children with severe PTSD symptoms, 50.0% had primary school education, 24.6% were secondary school graduates, 17.5% were high school graduates, 3.9% were literate, and 2.6% were university graduates. Of the parents of children with moderate PTSD, 48.5% were primary school graduates, 29.4% were middle school graduates, and 14.4% were high school graduates. The largest proportion of parents of children with mild PTSD (41.9%) were children with primary school graduates. When children’s post-traumatic stress levels were examined according to the damage their homes sustained after the earthquake, 74% of children with very severe PTSD had severely damaged homes, 14% had collapsed homes, and 10% had moderate damage. Of children with severe PTSD, 73.2% had severely damaged homes, 14.9% had collapsed homes, 8.3% had moderate damage, and 3.5% had minor damage. Of children with moderate PTSD, 67.5% had severely damaged homes, 22.7% had collapsed homes, 6.7% had moderate damage, and 2.6% had minor damage. Among children with mild PTSD, 67.7% had homes severely damaged, 19.4% were destroyed, 9.7% had moderate damage, and 3.2% had minor damage. Based on the children’s post-traumatic stress levels and the decrease in family income, the proportion of children with very severe PTSD was 50.0%, 46.1% with severe PTSD, and 56.7% with moderate PTSD. This proportion was found to be 45.2% for children with mild PTSD, and there was no statistically significant difference between the groups in terms of income loss (*p* > 0.05) (see Table 11).

**Table 11:** Comparison of the posttraumatic stress reaction scale for children according to sociodemographic variables

	No PTSD		Mild PTSD		Moderate PTSD		Severe PTSD		Very Severe PTSD		
	*n*	%	*n*	%	*n*	%	*n*	%	*n*	%	*p*
											-^a^
Gender	1	10.0	27	43.5	93	47.9	151	66.2	36	72.0	
Female	9	90.0	35	56.5	101	52.1	77	33.8	14	28.0	
Male											-^a^
Group											
12–18 age	10	1.8	62	11.5	194	35.7	228	41.9	50	9.1	
Time of Arrival to the Container Settlement											-^a^
≤ 1 month	5	50.0	39	62.9	114	58.8	134	58.8	26	52.0	
2–4 Months	4	40.0	19	30.6	55	28.4	84	36.8	22	44.0	
≥ 5 months	1	10.0	4	6.5	25	12.9	10	4.4	2	4.0	
Parental Education											-^a^
Illiterate	0	0.0	3	4.8	6	3.1	3	1.3	0	0.0	
Literate	0	0.0	2	3.2	7	3.6	9	3.9	1	2.0	
Primary School	2	20.0	26	41.9	94	48.5	114	50.0	22	44.0	
Secondary Scholl	6	60.0	19	30.6	57	29.4	56	24.6	19	38.0	
High School	1	10.0	7	11.3	28	14.4	40	17.5	5	10.0	
Unıversity Degree	1	10.0	5	8.1	2	1.0	6	2.6	3	6.0	
Job Loss											-^a^
Yes	4	40.0	28	45.2	110	56.7	108	47.4	27	54.0	
No	6	60.0	34	54.8	84	43.3	120	52.6	23	46.0	
Reduction in Income											0.208^*^
Yes	4	40.0	28	45.2	110	56.7	105	46.1	25	50.0	
No	6	60.0	34	54.8	84	43.3	123	53.9	25	50.0	
House Damage Status											-^a^
Destroyed	2	20.0	12	19.4	44	22.7	34	14.9	7	14.0	
Severely Damaged	6	60.0	42	67.7	131	67.5	167	73.2	37	74.0	
Moderately Damaged	2	20.0	6	9.7	13	6.7	19	8.3	5	10.0	
Slightly Damaged	0	0.0	2	3.2	5	2.6	8	3.5	0	0.0	
Damaged	0	0.0	0	0.0	1	0.5	0	0.0	1	2.0	
Household Size											-^a^
≤ 4	7	70.0	22	35.5	81	41.8	118	51.8	22	44.0	
> 4	3	30.0	40	64.5	113	58.2	110	48.2	28	56.0	
Type of School Attended											-^a^
Temporary Field School	3	30.0	17	29.3	48	27.0	54	24.4	13	26.5	
Regular School	7	70.0	41	70.7	125	70.2	164	74.2	36	73.5	
Open Education	0	0.0	0	0.0	5	2.8	3	1.4	0	0.0	

^a^*P*-value not reported because the assumptions of the Chi-square analysis were not met. *p* < 0.05 was considered statistically significant

*Chi-square analysis

## Discussion

Data on child health, such as morbidity, nutrition, and mortality, are international indicators that reflect not only overall health but also the level of development of countries. This study collected data on child health in container cities established as safe living spaces after the 2023 earthquake in Türkiye. This research is one of the limited studies that examines the health status of children aged 0–18 living in container settlements in Turkey after the 2023 earthquakes, based directly on field data and using a multidimensional approach. Considering that studies on planning and prioritizing child health services in the post-disaster period largely rely on secondary data or institutional records, this field study fills a significant gap in the literature. The findings provide evidence-based guidance for improving the quality of post-disaster child health services and developing needs-based interventions.

### Physical health

While acute food shortages can be quickly addressed by numerous aid organizations rapidly delivering aid to disaster-stricken areas, resulting in nutritional problems following disasters, it is not always possible to provide adequate and balanced nutrition in temporary settlements. Children in the growing and developing age group are among the most affected groups [17]. Our research found that a large proportion of children aged 0–2 continued to be breastfed both before and after the earthquake. However, some children experienced a decrease in breastfeeding frequency, most commonly due to a decrease in breast milk production, illness of the mother or baby, psychological stress caused by the disaster, and unsuitable environmental conditions, all of which disrupted breastfeeding. A study conducted in a temporary shelter after the 2010 Haiti earthquake also reported that breastfeeding was considered poor [22]. Studies in the literature also point to similar findings. In the post-disaster period, mothers reported difficulties breastfeeding due to reasons such as lack of privacy, lack of suitable breastfeeding spaces, inadequate social support, and mother-infant separation. It has been reported that mothers have difficulty breastfeeding their babies due to the presence of strangers or open spaces in temporary shelters [20–22]. Not only breastfeeding but also complementary feeding processes are severely affected by disaster conditions. Research results have shown that some families struggle to obtain formula and cannot ensure hygienic conditions for formula preparation. Difficulties accessing clean water and the unreliability of existing water sources make the use of ready-made formula risky. Similarly, it has been reported that mothers of children aged 0–2 years have difficulty obtaining formula, that existing formula causes digestive problems (food-related diarrhea/allergy problems) in some babies, and that hygienic formula preparation is difficult due to limited access to hot water heaters [20]. It has been reported that post-disaster emergency aid distribution of infant formula and commercial supplementary food was widespread without a preliminary needs assessment [62]. This suggests that the use of inappropriate formula can jeopardize child health. It is emphasized that simply providing formula is not sufficient for feeding infants and young children during disasters; the necessary materials for preparation, clean areas, and accurate instructions must also be provided [47].

Another notable finding of our research is that a significant portion of children did not receive any nutritional support in the post-disaster period. None of the participants were referred to a nutritionist, and the lack of professional support for parents demonstrates a serious lack of services related to post-disaster infant and young child nutrition. Similarly, Etiler’s (2012) assessment of post-disaster child health emphasizes the critical importance of essential micronutrient supplements, such as iron and zinc, for child health in the post-disaster period, but notes that these supplements were not systematically provided in the field [17]. A study conducted in Wechuan, China, found that the prevalence of anemia in children increased from 36.5% to 67.5% in the two years following the disaster, and that these children did not receive nutritional and micronutrient supplements [63].Following the 2023 Kahramanmaraş Earthquake, it was reported that inadequate nutritional content for the 0–3 age group, particularly due to problems with the supply of clean drinking water, uncontrolled and irregular distribution of unhealthy foods, and the repetition of the same types of foods, negatively impacted child nutrition. Research participants’ attention to inadequate protein intake and the almost complete absence of healthy options like fruit demonstrates that nutritional deprivation is problematic not only in terms of calories but also quality [24]. Compared to these studies in the literature, our results only provide data indicating that food supplementation was not taken. Further field research may reveal problems that arise from not taking food supplements. Early and long-term studies after the earthquake are therefore very important.

The nutrition of children aged 1–6 can often be overlooked during disasters, resulting in child nutrition neglect. This risk is even higher for children who have lost their families or been separated from their immediate environment [17, 64]. Research data indicates that children aged 3–11 experience more nutritional problems (unbalanced diet, loss of appetite, skipping meals, and difficulties accessing basic food groups) compared to adolescents in the post-disaster period. Among children aged 3–11 who experienced loss, 0.3% lost their fathers, 1.4% lost their siblings, and 50% lost other family members. Among adolescents, 0.3% lost their fathers, 0.2% lost their siblings, and 60.8% lost other family members. In Türkiye, it has been reported that children under 5 years of age, in particular, face significant difficulties accessing adequate and balanced nutrition during disasters, and that nutritional problems are more prevalent in this age group [64]. Similarly, following the 2015 Nepal Earthquake, the prevalence of malnutrition among school-aged children was high, and drinking water, hygiene, and sanitation conditions were deteriorated due to damage to urban infrastructure, which in turn increased rates of malnutrition in children [49]. This finding demonstrates that post-disaster nutrition is not limited to food but is closely linked to hygiene, water safety, and environmental conditions. Furthermore, the loss of regular meal patterns and school nutrition programs with the onset of schooling in this age group can be considered another factor negatively impacting children’s nutrition. Adolescents are more likely to have developed skills such as independent mobility, participation in food procurement, and food preferences, and therefore are considered to be at less risk of nutritional deficiencies in the post-disaster period than younger age groups. However, it should be noted that while adolescents appear to experience fewer nutritional problems in the post-disaster period, this does not necessarily mean that their needs are being adequately met. Because adolescence is a period of both increased growth and intensified psychological changes, it is clear that this age group also requires specialized nutritional support and monitoring after a disaster. Dhoubhadel et al. (2020) adopted a similar approach in their study conducted in temporary shelters in Nepal, emphasizing the critical importance not only of psychosocial support but also of uninterrupted and accessible nutritional support for the maintenance of healthy development of children [65]. Furthermore, they recommend increasing daily fruit and vegetable intake, ensuring dietary diversity, opting for micronutrient-enriched foods, and providing additional support if specific nutritional deficiencies are identified [29]. Consequently, post-disaster child nutrition services should be implemented with age-specific and holistic approaches, rather than standardized ones.

Monitoring anthropometric measurements is a critical indicator in assessing nutritional status during disasters. In this context, measuring body weight for height, which can be applied without requiring age, is one of the most fundamental parameters for assessing children’s nutritional status. Our study determined that there was an increase in children’s anthropometric measurements in the post-disaster period compared to the pre-disaster period. Studies conducted in different locations one year after the 2015 Nepal earthquake yielded different results regarding anthropometric data. While Thorne-Lyman et al. (2018) found no significant change in anthropometric measurements of children under five [66], Dhoubhadel et al. (2020) reported increased rates of stunting and underweight in children living in temporary shelters [65]. A study conducted in China found that two years after the 2008 Wenchuan Earthquake, despite the government providing 1,730 kcal of daily food support, rates of stunting and wasting remained high among children [63]. Studies conducted in Japan also demonstrate the emergence of multifaceted effects on post-disaster growth indicators. Significant changes in body mass index were detected at 42 months in infants exposed to the effects of the Great East Japan Earthquake. An increase in the prevalence of obesity and body mass index was observed in children who moved from temporary shelters to permanent housing [48]. This suggests that post-disaster nutrition interventions should consider not only calorie intake but also food quality and age-specific needs. While our nutritional data indicate inadequacy, the lack of a negative trend in anthropometric measurements highlights the need for further assessment of the situation through long-term studies.

Following a major disaster, the lack of clean tap water and inadequate toilet hygiene also harm the health of children in temporary shelters [33]. Our research found that children’s hygiene problems in the post-disaster period, the lack of clean room air, frequent water and electricity outages, and difficulties accessing hygiene supplies were among the main obstacles. These findings are consistent with reports from UNICEF highlighting the need to send large quantities of hygiene kits to disaster areas [13]. It was noted that access to clean water in evacuation shelters following the Japan earthquake and inadequate toilet hygiene negatively impacted the health of disaster victims [33]. Another study reported that many communities depended on external supplies for drinking water and hygiene, resorting to risky practices such as open defecation [50]. Similarly, logistical delays in the provision of hygiene supplies and increased risk of bacterial disease due to damaged water lines were noted in earthquakes in Türkiye [67]. A comparison of the 3–11 age group and the adolescent age group revealed that adolescent children are more able to maintain hygiene practices independently and regularly. Younger children, however, face more challenges due to factors such as access to water, crowded living spaces, and access to age-appropriate hygiene products. Our research data indicates that while bathing every other day is common for children, problems related to lice and oral hygiene are particularly pronounced in the younger age group. It has been reported that children living in container cities after the earthquake experienced significant difficulties meeting their hygiene, toilet, and bathing needs. They frequently became ill, and unhygienic conditions negatively impacted their physical health. Fecal accumulation and waste management issues caused widespread hygiene problems in their living spaces [68]. Post-earthquake studies reported a decrease in children’s toothbrushing habits, a negative impact on these habits due to changes in living environment, and a significant portion of children experienced interruptions in their oral care routines [69, 70]. The results demonstrate that post-disaster hygiene depends not only on physical resources but also on multidimensional factors such as age group, developmental needs, and logistical support. Supporting families, improving environmental infrastructure, and providing age-specific hygiene products are critical for meeting the hygiene needs of young children in particular.

Our country’s childhood vaccination calendar includes routine vaccinations for 13 diseases. These are diphtheria, pertussis, tetanus, polio, hepatitis B, hepatitis A, H. influenza type b, tuberculosis, measles, mumps, rubella, chickenpox, and pneumococcus [31]. Our research findings revealed that access to post-disaster immunization services varied significantly across age groups. Only one in 19 children aged 3–11 who were due for vaccination did not receive the vaccine, and only one in 5 children aged 12–18 who were due for vaccination did not receive the vaccine. While this rate demonstrates that health services are reaching adolescents to a large extent, the significantly lower overall vaccination card ownership in this group compared to other age groups suggests that these individuals may be at a disadvantage in terms of regular vaccination follow-up during the pre-disaster period. After the 2005 Pakistan earthquake, a large proportion of children completed their routine vaccinations; however, factors such as geographical accessibility, maternal lack of education, socioeconomic disadvantages, and ethnic identity negatively impacted the vaccination process [71]. Exposure to disasters has been reported to reduce the likelihood of timely vaccination in children [72]. Furthermore, research conducted by Kikuya et al. (2015) after the 2011 Tōhoku Earthquake in Japan indicates that parental attitudes and the child’s health status are important individual factors influencing vaccination decisions [27]. This suggests that the low vaccination rates among adolescents in our study may have been influenced not only by disaster conditions but also by parental involvement, knowledge, and attitudes. It has been reported that a large amount of childhood vaccines and antiserum were sent to disaster areas after the 2023 Kahramanmaraş-centered earthquakes in Türkiye [4]. This situation demonstrates that a rapid and organized response was given to maintain immunization services at the central level in our country. In this context, the effective monitoring and follow-up of vaccinations by health professionals, ensuring that missing vaccinations are completed, and preventing the emergence of vaccine-preventable diseases play a critical role in protecting the health of children and adolescents, especially in the post-disaster period.

Physical health, due to the impact of living conditions in container cities, has impacted disease development in children. In our study, the fact that the 2023 Türkiye earthquake occurred during the winter period is consistent with the prevalence of upper respiratory tract infections being the leading cause across all age groups. The discussion of hygiene and water problems in container cities in the preliminary data is consistent with the high prevalence of acute gastroenteritis, which is second, but there is no difference in prevalence across age groups. Skin diseases are third in all age groups, followed by eye infections and urinary tract infections. Due to factors such as poverty, limited resources, and inadequate infrastructure in developing countries, the risk of disease outbreaks after a disaster is extremely high. Mortality rates in temporary shelters after a disaster are 60 times higher than pre-disaster mortality rates. Factors such as prolonged overcrowding, poor hygiene habits, and immature immune systems increase children’s susceptibility to infectious diseases [17, 33]. Respiratory tract infections are generally identified as one of the most common health problems in children after a disaster. Wang et al. (2016) reported that 42.3% of admissions in children after the Nepal Earthquake were due to respiratory tract infections [26], while Ding et al. (2015) reported 47.3%. These data demonstrate that respiratory infections are a widespread global problem in post-disaster child health [25]. This finding is consistent with our findings, as skin infections were common in preschool children after the Nepal earthquake [26], and skin diseases and respiratory infections were reported as the second most common after the Lushan earthquake [25]. Following the Türkiye earthquake, the most common health problems in children under 5 were upper respiratory tract infections and diarrhea, while scabies was found to have clinical findings in 5.2% of children [50]. Following the major earthquake in Japan, reports of increased complaints such as influenza, itchy rashes, and eczema in children, and reports of increased allergic symptoms not only in the post-disaster period but also in the long term, suggest that disasters can have lasting effects on children’s immune systems [27]. These findings suggest that temporary housing conditions make children vulnerable to infections. Another study conducted on children referred to hospital after the February 6 Kahramanmaraş earthquake reported a rate of 12.5% ​​for urinary tract infections and 21.9% for skin and soft tissue infections [73]. Disaster conditions create a favorable environment for infectious diseases due to limited freshwater resources [74]. These data demonstrate the vital importance of access to clean water and sanitation services, especially for young children.

### Mental health

Because children in the early childhood years have immature verbal and emotional regulation skills, their psychological impacts manifest more at a behavioral level, highlighting their need for specialized support in the post-disaster period. At this point, the role of the caregivers surrounding the child becomes critical. In our study, when the post-earthquake mental health of children aged 0–2 was assessed, behavioral responses such as constant parental involvement, crying when separated, sleep disruption, and appearing anxious or confused about their surroundings were commonly observed. The literature also emphasizes that children in this age group generally respond to traumatic events through behavioral means. While early studies suggested that young children cannot cognitively understand trauma, later studies have revealed that infants and toddlers can also develop emotional and psychological responses to disasters [75, 76]. It has been reported that such behavioral symptoms are frequently observed in infancy in the post-disaster period, with reactions such as surprise, anxiety, reluctance to separate from parents, sleep disturbances, and constant crying being common. It is important for parents to manage their own anxiety and fears in the face of such emotional states experienced by infants and to maintain their child’s care routine as much as possible [36]. However, our study found that a large portion of parents of children aged 0–2 did not receive any psychological support. This suggests that not only the child but also the parent is deprived of psychosocial support after the disaster, and consequently, the child is indirectly at greater risk.

Among children aged 3 to 11, 51.7% experienced the loss of at least one family member in the earthquake. Our research examined the mental health of these children, noting a reluctance to leave their parents, difficulty transitioning to sleep, appearing anxious or uneasy about changes in their environment, asking intense questions about the earthquake, and some regressive behaviors (such as thumb sucking, bedwetting, and baby talk). It can be said that children responded to the trauma they experienced in a developmentally appropriate manner, and their search for psychological safety was prominent. However, social withdrawal behaviors such as avoiding friendships or refusing to go to school were not observed in children in this age group. On the contrary, a large portion of children maintained their desire to interact with their peers, but a significant increase in dependency behaviors toward their mothers was observed. Research supports the idea that some children exhibit excessive attachment to their parents after the disaster, while others develop resistance to rules and distrust of what their parents say [36,38,77 ]. In studies conducted in the 2–6 age group, children’s mental reactions to disaster were observed to include screaming, physical agitation, and aggression, along with emotional reactions such as surprise, fear, helplessness, restlessness, and sadness. A significant portion of children exhibit signs of traumatic arousal, such as being easily startled, and signs of re-experiencing, such as a sense of reliving the traumatic event, as well as post-traumatic stress symptoms such as nightmares, night terrors, and hyperarousal [51]. It has been reported that children experience intense fear and shock after a disaster, and that themes of anxiety and loss are prominent in their drawings [77]. It is also known that there is a close relationship between children’s psychological state and the mental health of their parents. Mizuki et al. (2021) found a significant correlation between mothers’ anxiety levels and children’s emotional disorders; Honda et al. (2019) demonstrated that post-traumatic stress symptoms observed in mothers after the Fukushima disaster had long-term effects on children’s mental health [78, 79]. Our study also found that a large portion of parents did not receive any psychological support. This suggests the need to develop holistic psychosocial support mechanisms not only for children but also for parents in the post-disaster period. It is recommended that children be supported in expressing their feelings after an earthquake, but without being forced to do so [80].

Our research findings reveal the mental health of the 12–18 age group using multidimensional data (general mental state, anxiety, and post-traumatic stress disorder). In the earthquake, 61.3% of adolescents experienced the loss of at least one family member. In our study, post-disaster internalized symptoms such as a desire for solitude, apathy, sleep disruption, and pessimism about the future were more prevalent among adolescent girls living in the container city, while externalizing reactions such as aggressive behavior, smoking/substance use, and decline in academic performance were more common in boys. These findings demonstrate that adolescents exposed to the disaster are severely affected not only physically but also psychosocially. Yorulmaz and Karadeniz (2021) emphasized symptoms such as attention problems, irritability, social withdrawal, and hopelessness seen in adolescents affected by the earthquake [36]. Research emphasizes that adolescents are affected in multiple ways due to environmental conditions, disruptions in the educational process, and virtual threats, as well as individual traumas. Even with the transition to distance education, loss of motivation, attention deficit, and anxiety disorders have been reported as common diagnoses [68, 81]. There is a need to evaluate the mental health of adolescents who are seen as a period of transition to adulthood more in depth. Our research results showed that, according to the results of the Spielberger State-Trait Anxiety Inventory, there were statistically significant differences between gender, the duration of arrival to the container city, and anxiety levels. It was determined that trait anxiety levels were higher in girls, while state anxiety scores were higher in boys. In this study, the state anxiety score was found to be 39.34 ± 5.94 in boys and 38.52 ± 5.45 in girls. In a study conducted after the Marmara Earthquake, it was reported that both state and trait anxiety levels in girls were higher than in boys [39]. Ertem et al. (2003) reported that 47.0% of the boys who experienced the earthquake had state anxiety and 80.6% had trait anxiety; girls reported that 81.4% had state anxiety and 47.9% had trait anxiety [82]. Similarly, in a study conducted by Karakaya et al. (2004) the mean state anxiety score of children was found to be 39.4 ± 10.5 and trait anxiety score was 44.61 ± 8.80; it was found that girls scored higher in all subscales [83]. A study by Sabuncuoğlu et al. (2003) also stated that girls reported significantly higher levels of state anxiety than boys [84]. Tang et al. (2020) reported that the anxiety level was significantly higher in girls than in boys in their study after the 2013 Yaan earthquake [85]. Our research results also show that trait anxiety was higher in children who arrived in the container city early, while state anxiety was more pronounced in those who arrived later. The loss of family members and the destruction of homes and living spaces pushed them to container life, and the subsequent difficulties of life in container cities affected anxiety levels. Mental health problems in adolescents aged 11–17 were associated with living in a severely affected area and exposure to post-earthquake trauma 18–31 months after the Nepal earthquake [83]. Post- disaster depression and depressive symptoms are reported in the long term as post-disaster post- traumatic stress disorder (PTSD) [38, 86]. The first response to post- earthquake mental health is to move children to a safe area and meet their nutritional and safety needs. It is recommended that pediatric psychological assessments be conducted at the initial encounter in hospitals and continued thereafter [80]. Furthermore, it is recommended that child-centered mental health services be integrated into emergency disaster programs [38].

Life in temporary shelters after an earthquake presents multifaceted challenges, especially for adolescents. Studies show that adolescents living in container cities experience serious psychosocial problems due to factors such as limited physical living space, lack of privacy, inadequate social support mechanisms, and an intense sense of uncertainty [87, 88]. This study found that rates of Child Post-Traumatic Stress Disorder (PTSD) in adolescents were as follows: 1.8% had no PTSD symptoms, 11.5% had mild PTSD, 35.7% had moderate PTSD, 41.9% had severe PTSD, and 9.1% had very severe PTSD symptoms. Furthermore, our results revealed that PTSD symptoms were significantly correlated with sociodemographic variables such as gender, duration of residence in the container city, parental education level, and house damage. PTSD levels were particularly high among girls, those whose homes were severely damaged, and those who settled in the container city early. This suggests that the severity of the trauma, along with the duration of exposure to violence, plays a determining role in psychological impact. Research demonstrates that PTSD symptoms are more common in girls and that their effects can last for a long time [41, 43, 44, 85]. A recent study conducted after the Kahramanmaraş earthquakes found very severe PTSD symptoms in 77% of children, and 71% of these symptoms were reported to be associated with symptoms of depression [52]. A study conducted three years after the 2008 Wenchuan earthquake in China by Pan et al. (2015) found that earthquake exposure, in which PTSD, depression, and anxiety symptoms were common, was associated with these symptoms [88]. Family loss, witnessing serious injury, and experiencing intense fear were identified as significant predictors of PTSD and anxiety. Some studies demonstrate the impacts on children’s health in the field, while others provide data on the causes and proposed solutions. Our research results provide detailed data on the physical and mental health status of children after the 2023 earthquake in Türkiye and contribute to the literature.

### Limitations and strengths

Our study has some limitations. The data collected from three container cities cannot be generalized to other container cities. This study, conducted immediately after the container cities were established, may have addressed the deficiencies affecting children’s health later, and the data covers the situation at that time. There is no data on the conditions under which the children lived before arriving at the container city, and the impact of living conditions during the acute phase, particularly on mental status, is a limitation.

Our research, conducted despite the challenges of applying post-earthquake research in container cities, sheds light on the period just after the earthquake. It is assumed that the study, conducted in different container cities and the large sample size, reflects the current state of child health in Hatay. Comparatively collecting child data across all age groups provides a powerful perspective, reflecting the situation across all age groups during the same period, despite the challenges of data interpretation and compilation.

## Conclusion

The study reflects health data for children aged 0–18 in temporary living centers, as an example from Hatay, following the 2023 earthquake. Data on children’s physical health includes nutrition, vaccinations, anthropometric measurements, hygiene and disease status, and mental health. Data on children’s physical health indicate nutritional deficiencies. Living conditions and hygiene deficiencies in container cities indicate illnesses, particularly acute respiratory infections and acute gastroenteritis, across all age groups. The mental health of children who lost family members and whose homes were damaged and who arrived in container cities was affected to varying degrees. Mental health data revealed symptoms of anxiety and varying levels of PTSD in the adolescent group. In light of these findings, it is recommended that health services for children living in temporary shelters be addressed within a preventive, monitoring, and empowering framework, moving away from an approach focused solely on disease treatment. Ensuring the continuity of nutrition and immunization services, implementing environmental regulations to improve hygiene conditions, and establishing regular monitoring programs for infectious diseases are crucial. Furthermore, considering the psychosocial impacts of disaster experiences on children, it is necessary to expand mental health screenings, counseling, and psychosocial support services, particularly for adolescents. It is suggested that long-term and holistic intervention programs be planned, with coordinated work between health, social services, and education sectors, to protect and improve children’s health after a disaster.

## Supplementary Information

Supplementary Material 1: Table S1. Distribution by Socio-Demographic Characteristics. Table S2. Height and Weight of Children Aged 0–18 Years by Gender. Table S3. Turkey’s 0–18 Years Immunization Schedule. Table S4. Hygiene Status of Children Aged 0–2 Years. Table S5. Mental Health Status of Children Aged 0–2 Years. Table S6. Mental Health Status of Children Aged 0–2 Years by gender. Table S7. Mental Health Status of Children Aged 3-11 Years. Table S8. Mental Health Status of Adolescents Aged 12–18 Years.

## Data Availability

The datasets generated and/or analyzed during the current study are available from the corresponding author upon reasonable request. The databases are not publicly avaliable.

## Abbreviations

PTSD: Child Post-Traumatic Stress Disorder
OECD: Organisation for Economic Co-operation and Development
OCHA: The United Nations Office for the Coordination of Humanitarian Affairs
UNICEF: United Nations International Children's Emergency Fund
TÜİK: Turkish Statistical Institute
