# Supplementary material for: Field study on the health status of children aged 0–18 in container cities after the 2023 Türkiye earthquake - Hatay example
Source: BMC Public Health. 2026 Mar 18;26:1367. doi: 10.1186/s12889-026-26945-w (PMC13112623; doi:10.1186/s12889-026-26945-w)
Supplement: Supplementary file 1 — Supplementary Material 1: Table S1. Distribution by Socio-Demographic Characteristics. Table S2. Height and Weight of Children Aged 0–18 Years by Gender. Table S3. Turkey’s 0–18 Years Immunization Schedule. Table S4. Hygiene Status of Children Aged 0–2 Years. Table S5. Mental Health Status of Children Aged 0–2 Years. Table S6. Mental Health Status of Children Aged 0–2 Years by gender. Table S7. Mental Health Status of Children Aged 3-11 Years. Table S8. Mental Health Status of Adolescents Aged 12–18 Years. [file 12889_2026_26945_MOESM1_ESM.docx]

**Supplementary Files**

**Supplemantary Table 1** Distribution by Socio-Demographic Characteristics

| **Variables** | **Frequency (n)** | **Percentage (%)** |
| --- | --- | --- |
| **Group** |  |  |
| 0-2 | 76 | 7.5 |
| 3-11 | 358 | 35.3 |
| 12-18 | 579 | 57.2 |
| **Container Cities** |  |  |
| Katar | 288 | 28.4 |
| Büyükdalyan | 658 | 65.0 |
| Palandöken | 67 | 6.6 |
| **Interviewed Parent** |  |  |
| Mother | 830 | 81.9 |
| Father | 142 | 14.1 |
| Other | 41 | 4.0 |
| **Parental Survival Status** | |  |
| Mother Alive / Father Deceased | 9 | 0.9 |
| Father Alive / Mother Deceased | 2 | 0.2 |
| Both Parents Alive | 1002 | 98.9 |
| **Parental Education** |  |  |
| Illiterate | 31 | 3.0 |
| Literate | 36 | 3.6 |
| Primary School | 415 | 41.0 |
| Secondary Scholl | 295 | 29.1 |
| High School | 191 | 18.9 |
| Unıversity Degree | 45 | 4.4 |
| **Parental Occupation** |  |  |
| Homemaker | 788 | 77.7 |
| Self- Employed | 73 | 7.2 |
| Laborer | 88 | 8.7 |
| Municipality Employee | 1 | 0.1 |
| Government Employee | 26 | 2.6 |
| Healthcare Worker | 11 | 1.1 |
| Accountant | 2 | 0.2 |
| Teacher | 3 | 0.3 |
| Bus Driver | 9 | 0.9 |
| Retired | 1 | 0.1 |
| Farmer | 7 | 0.7 |
| Unemployed | 4 | 0.4 |
| **Job Loss** |  |  |
| Yes | 515 | 50.8 |
| No | 498 | 49.2 |
| **Reduction in Income** |  |  |
| Yes | 513 | 50.6 |
| No | 500 | 49.4 |
| **House Damage Status** |  |  |
| Destroyed | 215 | 21.2 |
| Severely Damaged | 656 | 64.8 |
| Moderately Damaged | 79 | 7.8 |
| Slightly Damaged | 55 | 5.4 |
| Undamaged | 8 | 0.8 |
| **Time of Arrival to the Container Settlement** | |  |
| ≤ 1 month | 495 | 48.9 |
| 2-4 Months | 319 | 31.5 |
| ≥ 5 months | 199 | 19.6 |
| **Household Size** |  |  |
| ≤ 4 | 459 | 45.3 |
| > 4 | 554 | 54.7 |
| **Child’s Birth Order** |  |  |
| **1st** | 396 | 39.1 |
| 2nd | 372 | 36.7 |
| 3rd | 186 | 18.4 |
| 4th | 44 | 4.3 |
| 5th | 11 | 1.1 |
| 6th | 4 | 0.4 |
| **Child’s Age** | 11.23 ± 4.74 | |
| **Gender** |  |  |
| Female | 560 | 55.3 |
| Male | 453 | 44.7 |
| **School Attendance Status** |  |  |
| Yes | 815 | 87.0 |
| No | 122 | 13.0 |
| **Type of School Attended** |  |  |
| Temporary Field School | 325 | 39.9 |
| Regular School | 480 | 58.9 |
| Open Education | 10 | 1.2 |
| **Total** | 1013 | 100.0 |
|  |  |  |

**Supplemantary Table 2** Height and Weight of Children Aged 0–18 Years by Gender

|  | **Gender**  **Female** | **Male** |  |
| --- | --- | --- | --- |
| **Pre- Earthquake Weight** | 38.45 ± 16.43 | 40.14 ± 20.27 |  |
| **Post- Eathquake Weight** | 39.97 ± 16.39 | 42.51 ± 21.67 |  |
| **Pre- Earthquake Height** | 137.22 ± 28.00 | 138.28 ± 31.66 |  |
| **Post- Earthquake Height** | 139.36 ± 25.80 | 140.93 ± 29.38 |  |

**Supplementary Table 3** Turkey’s 0–18 Years Immunization Schedule

| **Age** | **Vaccines** |
| --- | --- |
| At birth | Hepatitis B (1st dose) |
| 2 months | BCG (Tuberculosis); Pneumococcal (PCV – 1st dose); 6-in-1 combination (DTaP-IPV-Hib-Hep B – 1st dose) |
| 4 months | Pneumococcal (PCV – 2nd dose); 6-in-1 combination (2nd dose) |
| 6 months | 6-in-1 combination (3rd dose); Oral Polio Vaccine (1st dose) |
| 12 months | Pneumococcal (booster); MMR (Measles, Mumps, Rubella); Varicella (Chickenpox) |
| 18 months | 6-in-1 combination (booster); Oral Polio Vaccine (2nd dose); Hepatitis A (1st dose) |
| 24 months | Hepatitis A (2nd dose) |
| 48 months | MMR (2nd dose); DTaP-IPV (booster) |
| 13 years | Td (Tetanus-Diphtheria booster) |
|  |  |

**Supplemantary Table 4** Hygiene Status of Children Aged 0–2 Years

|  | **Sample size**  **n** | | **Percentage**  **%** | |
| --- | --- | --- | --- | --- |
| **Maintaining Hygiene** |  | |  | |
| Yes | 46 | | 60.5 | |
| No | 30 | | 39.5 | |
| **Difficulty Accessing Water** |  | |  | |
| Yes | 1 | | 3.3 | |
| No | 29 | | 96.7 | |
| **Frequent Water Interruptions** |  | |  | |
| Yes | 4 | | 13.3 | |
| No | 26 | | 86.7 | |
| **Frequent Power Interruptions** |  | |  | |
| Yes | 3 | | 10.0 | |
| No | 27 | | 90.0 | |
| **Insufficient Hygiene Supplies** |  | |  | |
| Yes | 4 | | 13.3 | |
| No | 26 | | 86.7 | |
| **Inability to Maintain Clean Indoor Air Due to Structural Damage** | |  | |  |
| Yes | | 9 | | 30.0 |
| No | | 21 | | 70.0 |
| **Parental Hygiene Practices** | |  | |  |
| Yes | | 74 | | 97.4 |
| No | | 2 | | 2.6 |
| **Child’s Bathing Frequency** | |  | |  |
| Every day | | 18 | | 23.7 |
| Every 2 Days | | 39 | | 51.3 |
| Every 3 Days | | 19 | | 25.0 |
| **Provision of Age-Appropriate Shampoo** | |  | |  |
| Yes | | 73 | | 96.1 |
| No | | 3 | | 3.9 |
| **Ability to Maintain Clothing Cleanliness** | |  | |  |
| Yes | | 64 | | 84.2 |
| No | | 12 | | 15.8 |
| **Ability to Maintain Nail Cleanliness** | |  | |  |
| Yes | | 76 | | 100.0 |
| No | | 0 | | 0.0 |
| **Total** | | **76** | | **100.0** |

**Supplemantary Table 5** Mental Health Status of Children Aged 0–2 Years

|  | **Sample size**  **n** | **Percentage**  **%** |
| --- | --- | --- |
| **Loss of Family Members** |  |  |
| Yes | 38 | 50.0 |
| No | 38 | 50.0 |
| **Family Member Lost** |  |  |
| Sibling | 2 | 2.6 |
| Other Family Members | 36 | 47.4 |
| **Appearing Confused/Anxious** |  |  |
| Always | 7 | 9.2 |
| Sometimes | 61 | 80.3 |
| Never | 8 | 10.5 |
| **Wanting to Be Continuously with the Parent** |  |  |
| Always | 45 | 59.2 |
| Sometimes | 30 | 39.5 |
| Never | 1 | 1.3 |
| **Crying After Separation from the Parent** |  |  |
| Always | 37 | 48.7 |
| Sometimes | 38 | 50.0 |
| Never | 1 | 1.3 |
| **Change in Sleep Pattern** |  |  |
| Yes | 39 | 51.3 |
| No | 37 | 48.7 |
| **Receiving Psychological Support (Parent)** |  |  |
| Yes | 3 | 3.9 |
| No | 76 | 96.1 |
| **Total** | 76 | 100.0 |

**Supplementary Table 6** Mental Health Status of Children Aged 0–2 Years by gender

|  | **Female** | | **Male** | | **p** |
| --- | --- | --- | --- | --- | --- |
|  | **Sample size**  **n** | **Percentage**  **%** | **Sample size**  **n** | **Percentage**  **%** |  |
| **Loss of Family Members** |  |  |  |  | 0.645* |
| Yes | 20 | 47.6 | 18 | 52.9 |  |
| No | 22 | 52.4 | 16 | 47.1 |  |
| **Family Member Lost** |  |  |  |  | 1.00** |
| Sibling | 1 | 5.0 | 1 | 5.6 |  |
| Other Family Members | 19 | 95.0 | 17 | 94.4 |  |
| **Appearing Confused/Anxious** |  |  |  |  | -**^a^** |
| Always | 6 | 14.3 | 1 | 2.9 |  |
| Sometimes | 33 | 78.6 | 28 | 82.4 |  |
| Never | 3 | 7.1 | 5 | 14.7 |  |
| **Wanting to Be Continuously with the Parent** |  |  |  |  | -**^a^** |
| Always | 25 | 59.5 | 20 | 58.8 |  |
| Sometimes | 16 | 38.1 | 14 | 41.2 |  |
| Never | 1 | 2.4 | 0 | 0.0 |  |
| **Crying After Separation from the Parent** |  |  |  |  | -**^a^** |
| Always | 20 | 47.6 | 17 | 50.0 |  |
| Sometimes | 21 | 50.0 | 17 | 50.0 |  |
| Never | 1 | 2.4 | 0 | 0.0 |  |
| **Change in Sleep Pattern** |  |  |  |  | 0.474* |
| Yes | 20 | 47.6 | 19 | 55.9 |  |
| No | 22 | 52.4 | 15 | 44.1 |  |
| **Receiving Psychological Support (Parent)** |  |  |  |  | 0.248** |
| Yes | 3 | 7.1 | 0 | 0.0 |  |
| No | 39 | 92.9 | 34 | 100.0 |  |

*Chi-square analysis; **Fisher’s Exact test; ^a^P-value not reported because the assumptions of the Chi-square analysis were not met; p: Significance level; p < 0.05 considered statistically significant.

**Supplementary Table 7** Mental Health Status of Children Aged 3-11 Years

|  | **Sample size**  **n** | **Percentage**  **%** |
| --- | --- | --- |
| **Loss of Family Members** |  |  |
| Yes | 185 | 51.7 |
| No | 173 | 48.3 |
| **Family Member Lost** |  |  |
| Father | 1 | 0.3 |
| Sibling | 5 | 1.4 |
| Other Family Members | 179 | 50.0 |
| **Appearing Confused/Anxious** |  |  |
| Always | 58 | 16.2 |
| Sometimes | 276 | 77.1 |
| Never | 24 | 6.7 |
| **Wanting to Be Continuously with the Parent** |  |  |
| Always | 137 | 38.3 |
| Sometimes | 200 | 55.9 |
| Never | 21 | 5.9 |
| **Feeling Distrust Toward What the Parent Says** |  |  |
| Always | 14 | 3.9 |
| Sometimes | 126 | 35.2 |
| Never | 218 | 60.9 |
| Change in Sleep Pattern |  |  |
| Yes | 191 | 53.4 |
| No | 167 | 46.6 |
| **Waking Up Due to Nightmares** |  |  |
| Always | 35 | 9.8 |
| Sometimes | 194 | 54.2 |
| Never | 129 | 36.0 |
| **Asking Questions About the Earthquake** |  |  |
| Always | 84 | 23.5 |
| Sometimes | 210 | 58.7 |
| Never | 64 | 17.9 |
| **Exhibiting Avoidance Behaviors** |  |  |
| Always | 6 | 1.7 |
| Sometimes | 214 | 59.8 |
| Never | 138 | 38.5 |
| **Consistently Co-Sleeping with the Mother** |  |  |
| Yes | 197 | 55.0 |
| No | 23 | 6.4 |
| **Difficulty Making Friends** |  |  |
| Yes | 32 | 8.9 |
| No | 188 | 52.5 |
| **Refusing to Go to the Hospital When Necessary** |  |  |
| Yes | 7 | 2.0 |
| No | 213 | 59.5 |
| **Not Wanting to Go to School** |  |  |
| Yes | 69 | 19.3 |
| No | 151 | 42.2 |
| **Not Participating in Activities** |  |  |
| Yes | 17 | 4.7 |
| No | 203 | 56.7 |
| **Exhibiting Regression Symptoms** |  |  |
| Yes | 97 | 27.1 |
| No | 261 | 72.9 |
| **Thumb Sucking** |  |  |
| Yes | 22 | 6.1 |
| No | 75 | 20.9 |
| **Enuresis** |  |  |
| **Yes** | 54 | 55.7 |
| No | 43 | 12.0 |
| **Crawling** |  |  |
| **Yes** | 5 | 1.4 |
| No | 92 | 25.7 |
| **Baby Talk** |  |  |
| Yes | 18 | 5.0 |
| No | 79 | 22.1 |
| **Not Eating Independently** |  |  |
| Yes | 3 | 0.8 |
| No | 94 | 26.3 |
| **Wanting to Be Carried Constantly** |  |  |
| Yes | 7 | 2.0 |
| No | 90 | 25.1 |
| **Speech Regression** |  |  |
| Yes | 3 | 0.8 |
| No | 94 | 26.3 |
| **Wanting a Pacifier** |  |  |
| Yes | 17 | 4.7 |
| No | 80 | 22.3 |
| **Receiving Psychological Support (Parent)** |  |  |
| Yes | 20 | 5.6 |
| No | 338 | 94.4 |
| **Total** | 358 | 100.0 |

**Supplementary Table 8** Mental Health Status of Adolescents Aged 12–18 Years

|  | **Sample size**  **n** | **Percentage**  **%** |
| --- | --- | --- |
| **Loss of Family Members** |  |  |
| Yes | **355** | **61.3** |
| No | 224 | 38.7 |
| **Family Member Lost** |  |  |
| Father | 2 | 0.3 |
| Sibling | 1 | 0.2 |
| Other Family Members | 352 | 60.8 |
| **Appearing Confused/Anxious** |  |  |
| Always | 31 | 5.4 |
| Sometimes | 514 | 88.8 |
| Never | 34 | 5.9 |
| **Wanting to Be Alone Constantly** |  |  |
| Always | 41 | 7.1 |
| Sometimes | 361 | 62.3 |
| Never | 177 | 30.6 |
| **Experiencing Lack of Interest in Normal Activities** |  |  |
| Always | 46 | 7.9 |
| Sometimes | 374 | 64.6 |
| Never | 159 | 27.5 |
| **Having Negative Thoughts About the Future** |  |  |
| Always | 123 | 21.2 |
| Sometimes | 388 | 67.0 |
| Never | 68 | 11.7 |
| **Change in Sleep Pattern** |  |  |
| Yes | 189 | 32.6 |
| No | 390 | 67.4 |
| **Waking Up Due to Nightmares** |  |  |
| Always | 40 | 6.9 |
| Sometimes | 333 | 57.5 |
| Never | 206 | 35.6 |
| **Substance or Cigarette Use During This Period** |  |  |
| Yes | 15 | 2.6 |
| No | 564 | 97.4 |
| **Experiencing Lack of Interest in School** |  |  |
| Yes | 325 | 56.1 |
| No | 254 | 43.9 |
| **Decline in School Performance** |  |  |
| Yes | 250 | 43.2 |
| No | 329 | 56.8 |
| **Exhibiting Aggressive Behaviors** |  |  |
| Yes | 67 | 11.6 |
| No | 512 | 88.4 |
| **Receiving Psychological Support (Child)** |  |  |
| Yes | 54 | 9.3 |
| No | 525 | 90.7 |
